# Discovery of Potent VEGFR-2 Inhibitors based on Furopyrimidine and Thienopyrimidne Scaffolds as Cancer Targeting Agents

**DOI:** 10.1038/srep24460

**Published:** 2016-04-15

**Authors:** Marwa A. Aziz, Rabah A. T. Serya, Deena S. Lasheen, Amal Kamal Abdel-Aziz, Ahmed Esmat, Ahmed M. Mansour, Abdel Nasser B. Singab, Khaled A. M. Abouzid

**Affiliations:** 1 Pharmaceutical Chemistry Department, Faculty of Pharmacy, Ain Shams University, Abassia, Cairo 11566, Egypt; 2 Department of Pharmacology and Toxicology, Faculty of Pharmacy, Ain Shams University, Abassia, Cairo 11566, Egypt; 3 Department of Pharmacology and Toxicology, Faculty of Pharmacy, Al-Azhar University, Cairo, Egypt; 4 Pharmacognosy Department, Faculty of Pharmacy, Ain Shams University, Abassia, Cairo 11566, Egypt; 5 Center for Drug Discovery and Development Research, Faculty of Pharmacy, Ain Shams University, Abassia, Cairo 11566, Egypt

## Abstract

Vascular endothelial growth factor receptor-2 (VEGFR-2) plays a crucial role in cancer angiogenesis. In this study, a series of novel furo[2,3-*d*]pyrimidine and thieno[2,3-*d*]pyrimidine based-derivatives were designed and synthesized as VEGFR-2 inhibitors, in accordance to the structure activity relationship (SAR) studies of known type II VEGFR-2 inhibitors. The synthesized compounds were evaluated for their ability to *in vitro* inhibit VEGFR-2 kinase enzyme. Seven compounds **(15b, 16c, 16e, 21a, 21b, 21c** and **21e)** demonstrated highly potent dose-related VEGFR-2 inhibition with IC_50_ values in nanomolar range, of which the thieno[2,3-*d*]pyrimidine based-derivatives **(21b, 21c** and **21e)** exhibited IC_*50*_ values of 33.4, 47.0 and 21 nM respectively. Moreover, furo[2,3-*d*]pyrimidine-based derivative **(15b)** showed the strongest inhibition of human umbilical vein endothelial cells (HUVEC) proliferation with 99.5% inhibition at 10 μM concentration. Consistent with our *in vitro* findings, compounds (**21b** and **21e)** orally administered at 5 and 10 mg/kg/day for 8 consecutive days demonstrated potent anticancer activity in Erhlich ascites carcinoma (EAC) solid tumor murine model. Such compounds blunted angiogenesis in EAC as evidenced by reduced percent microvessel via decreasing VEGFR-2 phosphorylation with subsequent induction of apoptotic machinery. Furthermore, Miles vascular permeability assay confirmed their antiangiogenic effects *in vivo*. Intriguingly, such compounds showed no obvious toxicity.

It has become increasingly certain that angiogenesis, or new blood vessel formation, plays a central role in the cancer development^1^. The sprouting of new blood vessels allows for the growth of local tumors, offers them a royal road to other parts of the body and feeds their growth in distant sites, hence enables malignant cells to escape from the primary tumor, to enter into circulation and establish distant metastasis elsewhere^2^. A finely tuned equilibrium between anti- and pro-angiogenic molecules modulates the complex and dynamic events during angiogenesis^3^. One of the most specific and crucial regulators of angiogenesis is the vascular endothelial growth factor family (VEGFs)^4^. They exert their biologic effects through interaction with the kinase domain of Vascular Endothelial Growth Factor Receptors (VEGFRs 1–3). Upon binding to the extracellular domain of the receptor, they activate a cascade of downstream signaling pathways through the dimerization and autophosphorylation of the intracellular receptor tyrosine kinases^5^. VEGFR-2 represents a major target within the angiogenesis-related kinases, hence considered the most important transducer of VEGF-dependent angiogenesis^6^. Thus, inhibition of VEGF/VEGFR signaling pathway is regarded as an attractive therapeutic target for inhibition of tumor angiogenesis and subsequent tumor growth^7^. This inhibition has been achieved through two main approaches. First, by blocking ligand binding to the extracellular domain of the kinase receptor with monoclonal antibodies. Bevacizumab (Avastin^®^) is a humanized monoclonal antibody that specifically blocks the interaction of VEGF ligands to VEGFR-2^6^. In 2004, it was approved by FDA for the treatment of metastatic colorectal cancer^8^ and then for breast^9^ and lung cancers^10^ as well. The second approach to block the VEGF pathway is achieved by preventing the activation of VEGFR-2 receptors using tyrosine kinase inhibitors (RTKIs)^11^. The most advanced inhibitors are Sunitinib **(I)** (with IC_*50*_ = 10 nM) which is FDA approved for treatment of gastrointestinal stromal tumor^12^ and advanced renal cell carcinoma^13^. Sorafenib **(II)** (with IC_*50*_ = 90 nM) has been approved for patients with metastatic renal cell carcinoma^14^, and unrespectable hepatocellular carcinoma^15^ (Fig. 1).

Kinase inhibitors can be classified into two major categories. Type I inhibitors mainly recognize the active conformation of VEGFR-2, just binding in and around the region originally occupied by the adenine ring of ATP. For type II kinase inhibitors, they stabilize the DFG-out (inactive) conformation of the enzyme which occurs by movement of the DFG motif (Aspartate- Phenylalanine- Glycine motif). This enables them to occupy a hydrophobic site, usually called allosteric site, created by the new rearrangement and directly adjacent to the ATP binding pocket. This allosteric site is only revealed in the inactive DFG-out conformation of the kinase^16^. Type-II kinase inhibitors have several advantages over type-I inhibitors, including improved kinase selectivity and slower off-rates^17^.

## Rationale and Design

**Figure 1:**
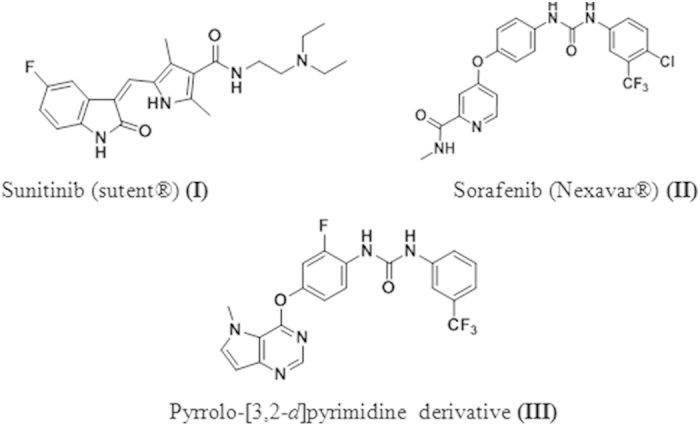
Some VEGFR-2 inhibitors currently approved or in clinical trials.

Study of the structure activity relationships (SAR) and common pharmacophoric features shared by various VEGFR-2 inhibitors, as well as analysis of binding modes of sorafenib **(II)** (PDB code **4ASD**)^18^ and pyrrolo-[3,2-*d*]pyrimidine derivative **(III)** (Fig. 1) (IC_*50*_ = 6.2 nM) (PDB code **3VHE**)^19^ revealed that most type II VEGFR-2 inhibitors shared three main features^20^. (1) The core structure of most inhibitors consists of a flat hetero aromatic ring system that occupies the ATP binding region and binds to the hinge region via an essential hydrogen bond with the backbone NH of Cys919 residue. (2) Most inhibitors have hydrogen bond donor–acceptor pair, which is either an amide or urea moiety, aiming interaction with Glu88 and Asp1046 residues in the DFG domain of the enzyme. The NH motifs of the urea or amide moiety usually form two hydrogen bonds with Glu885, whereas the CO motifs form another hydrogen bond with Asp1046. (3) The terminal aryl moiety of the inhibitors occupy the newly created allosteric hydrophobic pocket revealed when the phenylalanine residue of the DFG loop flips out of its lipophilic pocket defining DFG-out or inactive conformation. Thus, hydrophobic interactions are usually attained in this allosteric binding region^21^. Furthermore, analysis of the X-ray structure of various inhibitors bound to VEGFR-2 confirmed the sufficient space available for various substituents around the terminal aromatic ring^22^.

**Figure 2:**
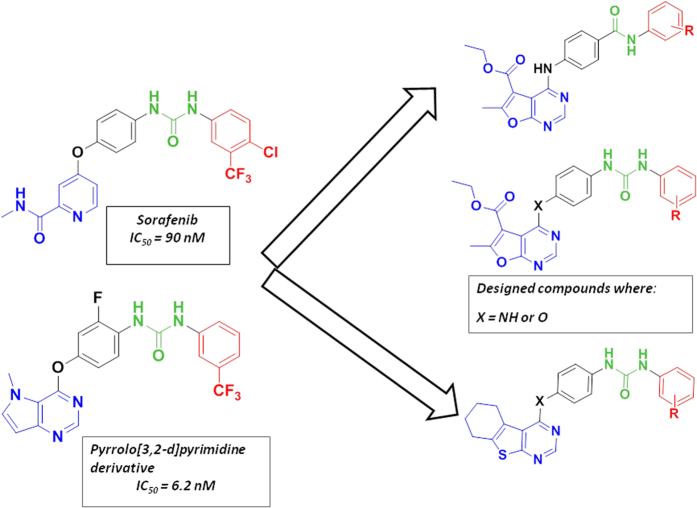
Essential pharmacophoric features of type II inhibitors of the designed compounds revealing the main scaffolds in blue. The urea or amide linkers are presented in green color and finally the terminal substituted aromatic moiety occupying the allosteric hydrophobic pocket is presented in red color.

Based on the above study and via application of bioisosteric modification strategies, two series of furo[2,3-*d*]pyrimidine and thieno[2,3-*d*]pyrimidine based-derivatives were designed and synthesized, to act as type II VEGFR-2 inhibitors. Furo[2,3-*d*]pyrimidine and thieno[2,3-*d*]pyrimidine scaffolds were introduced as scaffolds that are not extensively reported as VEGFR inhibitors. The two series were designed bearing the aforementioned essential pharmacophoric fragments for type-II inhibitors, aiming to maintain the same binding interactions between the N-1 nitrogen of the fused pyrimidine scaffolds with the Cys919 NH in the hinge region, and between the hydrogen bond donor–acceptor pair represented by amide or urea moiety with Glu885 and Asp1046 residues. In addition, the terminal aromatic ring substituted with various lipophilic groups (R), was designed to occupy the back hydrophobic pocket (Fig. 2).

## Results and Discussion

### Chemistry

**Figure 3:**
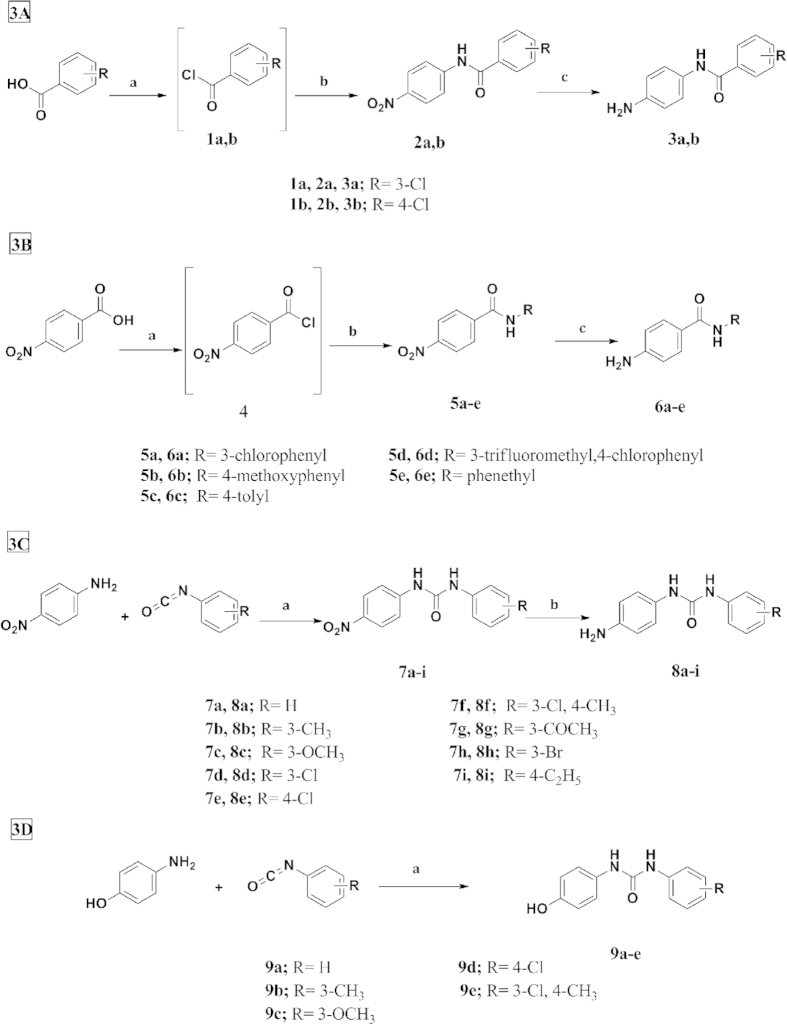
(**A**) Synthesis of N-(4-aminophenyl)-substituted benzamides. Reagents and conditions: a) SOCl_2_, reflux, 2–3 hrs; (**B**) p-nitroaniline, DCM, rt, 24 hrs; (**c**) H_2_, Pd/C, EtOH, 30 min. (**B**) Synthesis of 4-Amino-N-substituted benzamides. Reagents and conditions: (**A**) SOCl_2_, reflux, 7 hrs; (**B**) Aniline derivatives, DCM, rt, 2–24 hrs; (**c**) H_2_, Pd/C, EtOH, 30 min. (**C**) Synthesis of 1-(4-Aminophenyl)-3-substituted phenylureas. Reagents and conditions: (**A**) DCM, rt, 24 hrs; (**B**) H_2_, Pd/C, MeOH, 30 min. (**D)** Synthesis of 1-(4-Hydroxyphenyl)-3-substituted phenylureas. Reagents and conditions: (**A**) Dioxane, rt, 1 hour.

The various key intermediates and final compounds were synthesized according to the general pathways described in Schemes 1–3. Final compounds incorporating substituted amide and urea moieties were obtained utilizing the corresponding intermediates **(3a,b, 6a–e, 8a–i, 9a–e),** which were synthesized according to the routes outlined in Schemes 1a–d. Carboxylic acid derivatives were activated through their reaction with thionyl chloride^23^ to give acyl chlorides **(1a,b)**, which upon reaction with *p*-nitroaniline in dry DCM in the presence of TEA^24^ formed the amides **(2a,b)**. The nitro derivatives **(2a,b)** were reduced to their corresponding amines by Pd/C-catalyzed hydrogenation in ethanol^25^ to give N-(4-aminophenyl)-substituted benzamides **(3a,b)** (Fig. 3A). On the other hand, *p*-nitrobenzoic acid was activated using thionyl chloride to afford 4-nitrobenzoyl chloride **(4)**^26^, which was reacted with different anilines^24^ to form the amides **(5a–e).** Using palladium charcoal as the catalyst, the reduction of **5a–e** was performed in ethanol^25^ to give 4-Amino-N-substituted benzamides (**6a–e)** (Fig. 3B). The urea derivatives **(8a–i)** were prepared by reacting *p*-nitroaniline with the appropriate isocyanate in dry DCM for 24 hours^27^ to afford compounds **(7a–i)**, which were reduced to their corresponding amino derivatives using 10% Pd-C in methanol^28^ to give the 4-Aminophenyl substituted phenylureas **(8a–i)** (Fig. 3C). While the synthesis of 4-hydroxyphenyl-substitutedphenylureas **(9a–e)** was accomplished by stirring *p*-aminophenol with various isocyanates in dry dioxane at room temperature for 1 hour^29^ affording compounds **(9a–e)** (Fig. 3D).

**Figure 4:**
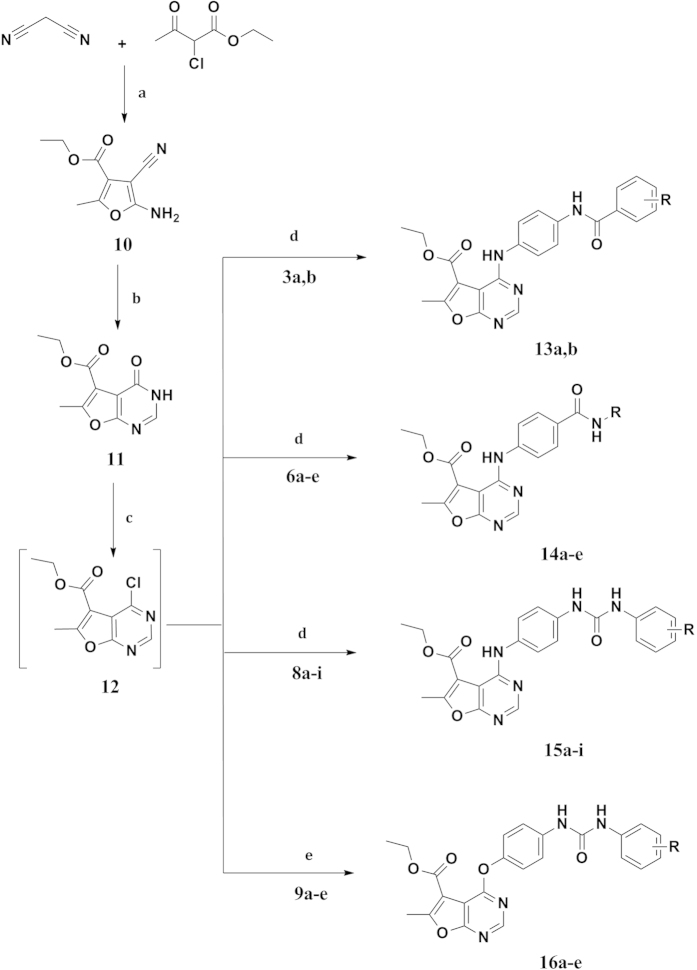
Synthesis of Ethyl 4-substituted-6-methylfuro[2,3-*d*]pyrimidine-5-carboxylate Reagents and conditions. (**a**) NaOEt, rt, 6 hrs; (**b**) HCOOH, acetic anhydride, reflux, 48 hrs; (**c**) POCl_3_, reflux, 3 hrs; (**d**) Ethanol, reflux, 18–48 hrs; (**e**) Acetonitrile, Cs_2_CO_3_, 60 ^o^C, 3–4 hrs.

The synthesis of furo[2,3-*d*]pyrimidine target structures (Fig. 4) was started with the preparation of the furan derivative **(10)** by reacting ethyl 2-chloroacetoacetate with malononitrile in Na ethoxide according to the reported method^30^. Cyclization of **10** was afforded via its reflux with formic acid and acetic anhydride for 48 hours^31^ to provide the furo[2,3-*d*]pyrimidinone derivative **(11).** Chlorination of **11** with POCl_3_^32^, followed by coupling with the key intermediates **(3a,c, 6a–e, 8a–i** and **9a–e)**, afforded the furo[2,3-*d*]pyrimidine-based compounds **(13a,b, 14a–e, 15a–i, 16a–e)** respectively.

**Figure 5:**
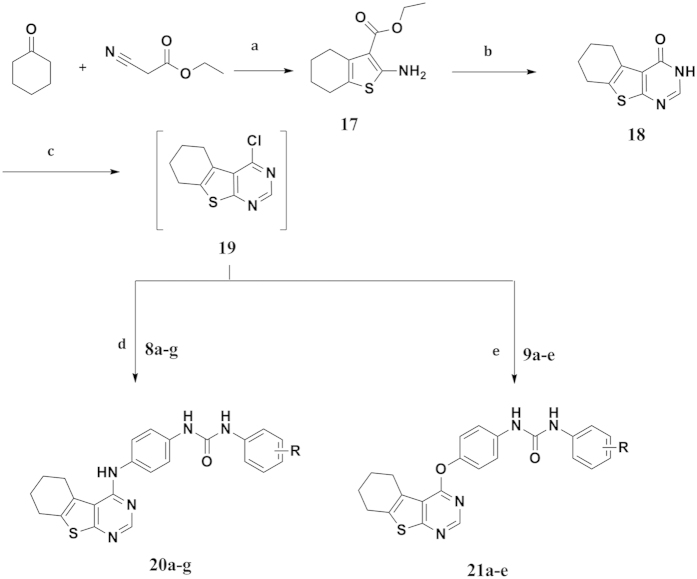
Synthesis of 4-substituted-5,6,7,8-tetrahydrobenzo[4,5]thieno[2,3-*d*]pyrimidine. Reagents and conditions: (**a**) S, piperidine, W.B at 50–60 ^o^C, 16 hrs; (**b**) HCONH_2_, reflux, 3 hrs; (**c**) POCl_3_, reflux, 3 hrs; (**d**) Ethanol, reflux, 24 hrs; (**e**) Acetonitrile, Cs_2_CO_3_, 60 ^o^C, 6 hrs.

Finally, the synthesis of thieno[2,3-*d*]pyrimidine target compounds (Fig. 5) was initiated via the preparation of intermediate **(17)**, applying Gewald reaction^33^–, which was then cyclized by formamide^34^ to afford the thieno[2,3-*d*]pyrimidinone **(18)**. Chlorination of **18** by POCl_3_^35^, followed by reaction of **19** with the urea derivatives **(8a–g** and **9a–e)** afforded the final target compounds **(20a–g** and **21a–e)**.

### Biological Evaluation

**Figure 6:**
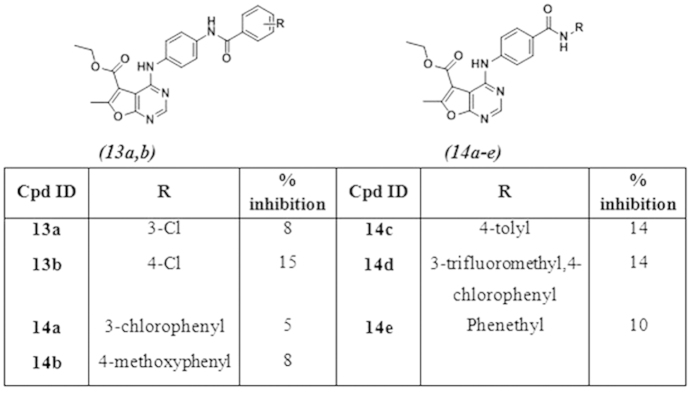
Percent inhibition of VEGFR-2 enzymatic activity exhibited by the target amide-based compounds *(13a,b, 14a–e)* at 10 μM.

**Figure 7:**
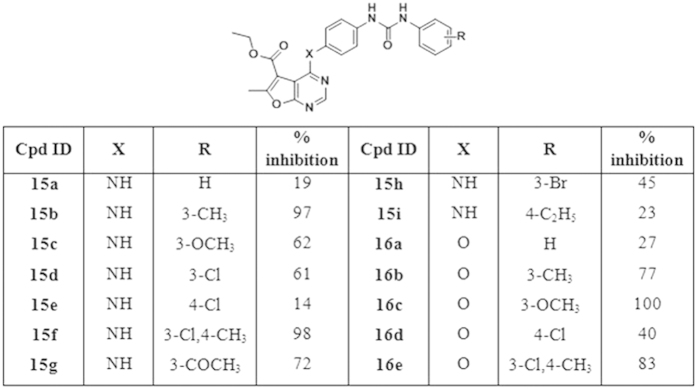
Percent inhibition of VEGFR-2 enzymatic activity achieved by the target biarylureas linked to furo[2,3-*d*]pyrimidine core *(15a–i, 16a–e)* at 10 μM.

**Figure 8:**
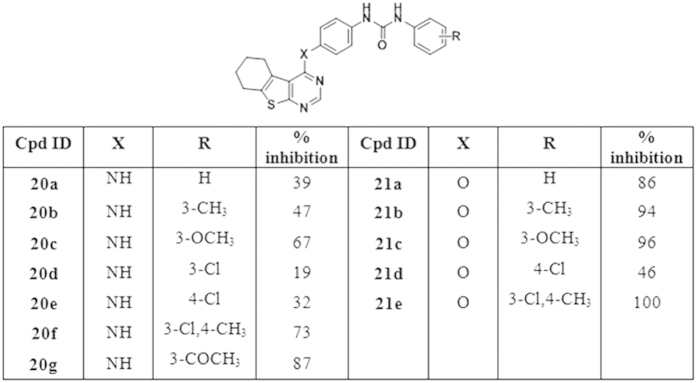
Percent inhibition of VEGFR-2 enzymatic activity achieved by the target biarylureas linked to thieno[2,3-*d*]pyrimidine core *(20a–g, 21a–e)* at 10 μM.

*In vitro VEGFR-2 tyrosine kinase activity*. Initial screening at single dose of 10 μM concentration: The VEGFR-2 tyrosine kinase assays were performed at BPS Bioscience, San Diego, CA, USA (www.bpsbioscience.com). In an initial screening; all synthesized final compounds were evaluated for their inhibitory activity against VEGFR-2 kinase at a single dose concentration of 10 μM. At this concentration, the furo[2,3-*d*]pyrimidine derivative **(16c)** and the thieno[2,3-*d*]pyrimidine derivative **(21e)**, both incorporating a substituted biarylurea motif linked via an ether linker to the parent scaffold, have demonstrated a potent inhibition of 100% for the VEGFR-2 kinase activity. Nevertheless, significant inhibition above 70% was also exhibited by several other investigated compounds namely **(15b, 15f, 15g, 16b, 16c, 16e, 20f, 20g, 21a, 21b, 21c and 21e)**. The mean percent VEGFR-2 inhibition of the investigated compounds on VEGFR-2 at 10 μM concentration are shown in Figs 6,7 and 8. (Sorafenib as a lead compound showed 95% VEGFR-2 inhibition)^36^.

Structure activity relationship among the newly synthesized furo[2,3-*d*]pyrimidine and thieno[2,3-*d*]pyrimidine derivatives has been closely investigated. As compared to the weak inhibitory activity (5–15%) exhibited by the amide derivatives **(13a,b, 14a–e)** at 10 μM concentration (Fig. 6), it was revealed that the incorporation of the biarylurea moiety in compounds **(15a–i, 16a–e, 20a–g, 21a–e)** resulted in significant increase in enzymatic activity in both of the fused pyrimidine series.

Further comparison of the VEGFR-2 inhibitory activity among the biarylurea derivatives revealed that the replacement of NH linker by an ether linker at 4-position of the fused pyrimidine core generally resulted in higher levels of enzymatic inhibition. **(15a,c,e** & **20a,b,c,e,f** compared to **16a,c,d** & **21a–e)** (Figs 7 and 8).

Systematic investigation of various substituents incorporated in the terminal phenyl ring revealed the relatively weak activity exhibited in cases of unsubstituted terminal phenyl ring **(15a, 16a, 20a)**, which was consistent with the previously reported SAR^22^. However, incorporation of different substituents, specially at 3-position of the terminal phenyl ring was generally well tolerated and resulted in considerable increase in inhibitory activity in both fused pyrimidine series of compounds. This was revealed in the results of the 3-CH_3_ derivatives **(15b, 16b, 21b)**, the 3-OCH_3_ **(16c**, **20c, 21c)**, the 3-COCH_3_ **(15g, 20g)** as well as the disubstituted 3-Cl, 4-CH_3_ derivatives **(15f, 16e, 20f, 21e)** which exhibited the highest inhibition percent ranging from 72–100% at 10 μM concentration. Surprisingly, the 3-Cl derivatives **(15d, 20d)** and 3-Br derivative **(15h)** showed weak to moderate inhibition percent of 61, 19 and 45% respectively. On the other hand, the 4-Cl **(15e, 16d, 20e, 21d)** and 4-C_2_H_5_ derivatives **(15i)** exhibited weaker inhibitory activity ranging from 14–46% at the same concentration.

Evaluation of potential enzyme inhibitory activity (IC_50_): Promising candidates, which exhibited VEGFR-2 inhibition percent above 75% at 10 μM concentration **(15b, 15f, 16b, 16c, 16e, 20g, 21a, 21b, 21c, 21e),** were further investigated for their dose-related VEGFR-2 enzymatic inhibition at 5 different concentrations (1 nM, 10 nM, 100 nM, 1 μM, 10 μM) to subsequently calculate their IC_*50*_ values (Table 1). Most of the investigated compounds exhibited potent VEGFR-2 inhibitory activity with IC_*50*_values in nanomolar range. The thieno[2,3-*d*]pyrimidine-based derivative **(21e)** linked to the 1-(3-chloro-4-methylphenyl)-3-phenyl urea tail via an ether linkage, showed highly potent single-digit nanomolar VEGFR-2 inhibition (IC_*50*_ of 21 nM).

*In vitro* multiple-kinase inhibition assay. Multiple-kinase inhibition assay was carried out to evaluate the effect of the most potent compounds ***(16e, 21b, 21c, 21e)*** on other selected kinases such as c-Kit, c-Raf, c-Src and RET kinases. Kinase enzymatic activity of the tested compounds was evaluated against a reference kinase inhibitor at 10 μM (Table 2).

These kinases were chosen for assay as they are potently inhibited by the lead compound sorafenib. It inhibits several members of the RAF/MEK/ERK signaling cascade, including serine/threonine kinases c-Raf (IC_*50*_ = 6 nM) and wild-type b-Raf (IC_*50*_ = 25 nM)^37^. In addition to VEGFR-1/2/3 (IC_*50*_ = 26 nM, IC_*50*_ = 90 nM, and IC_*50*_ = 20 nM, respectively), sorafenib inhibits multiple pro-angiogenic receptor tyrosine kinases, including platelet-derived growth factor receptor-B (PDGFR-B) (IC_*50*_ = 57 nM), stem cell factor receptor (c-KIT) (IC_*50*_ = 68 nM), fms-related tyrosine kinase 3 receptor (FLT3) (IC_*50*_ = 33 nM), fibroblast growth factor receptor 1 (FGFR1) (IC_*50*_ = 580 nM)^37^, and RET tyrosine kinase (IC_*50*_ = 50 nM)^38^.

The four tested compounds exhibited good to potent enzymatic inhibition percent against c-kit, RET kinases ranging from 64–91%. They also showed moderate activity against c-Raf ranging from 26–71%. On the other hand, the four compounds showed weak inhibitory activity against c-Src kinase ranging from 1–8% (Table 2). These data suggest that these compounds display significant inhibition on other pro-angiogenic receptor kinases besides their VEGFR-2 inhibition.

*In vitro HUVEC Anti-proliferative assay.* The HUVEC cell line Anti-proliferative assay for selected compounds was also carried out in BPS Bioscience Corporation, San Diego, CA, USA (www.bpsbioscience.com).

Angiogenesis process involves endothelial cell (EC) sprouting from the parent vessel, followed by migration, proliferation, alignment, tube formation, and anastomosis to other vessels. Several *in vitro* models have attempted to recreate this complex sequence of events^39^. Human umbilical vein endothelial cells (HUVECs) have played a major role as a model system for the study of the regulation of endothelial cell function and the role of the endothelium in the response of the blood vessel wall to stretch, shear forces, and the development of atherosclerotic plaques and angiogenesis. Most endothelial cell assays utilize human umbilical vein endothelial cells (HUVECs) or bovine aortic endothelial cells (BAECs) being good representatives of vascular endothelial cells *in vivo*, besides being relatively easy to harvest from large blood vessels^40^.

**Figure 9:**
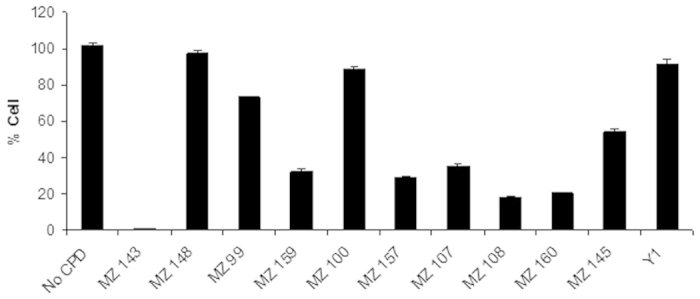
The bar graphs show the HUVECs growth percentage after treatment with the target compounds.

Ten compounds **(15b, 15f, 16b, 16c, 16e, 20g, 21a, 21b, 21c, 21e)**, exhibiting VEGFR-2 inhibition percent more than 75%, were selected to be tested for their ability to *in vitro* inhibit HUVEC cell line proliferation, using doxorubicin as control. The results are illustrated in Table 3 and Fig. 9.

The given test compounds manifested a varied anti-proliferative activity against HUVEC cell line. Compound **(15b)** (VEGFR-2 IC_*50*_ = 946 nM) showed the highest growth inhibition percent (99.50%). In addition, compounds **(21b, 21c)** (VEGFR-2 IC_*50*_ = 33.4 and 47.0 nM respectively) also manifested significant inhibition of HUVEC cell lines of 81.97 and 79.15% respectively.

However, despite their potent VEGFR-2 inhibitory activity, compounds **(15f, 16b, 16e, 21e)** exhibited low to moderate anti proliferative activity against HUVEC cell line.

## Molecular docking study

### Docking study

**Figure 17:**
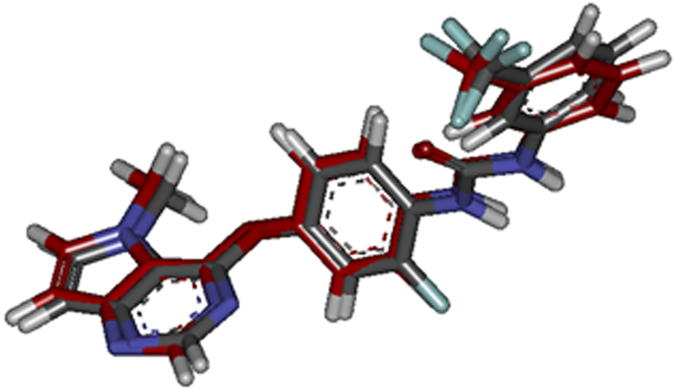
The alignment between the bioactive conformer (colored in red) and the docked pose of the same compound at VEGFR-2 binding site.

Molecular docking investigational study was performed in order to gain further insight into the binding modes and orientations of the synthesized compounds into the ATP binding site of VEGFR-2 kinase enzyme. Docking study was performed using C-Docker protocol in Discovery Studio 2.5 Software. The crystal structure of VEGFR-2 complexed with its pyrimidine-based inhibitor **(III)** was obtained (PDB code 3VHE)^19^. It presents the enzyme in its ‘DFG-out’ inactive conformation. Validation of docking algorithm was achieved by redocking the lead compound **(III)** into the active site of VEGFR-2 (3VHE) using C-Docker algorithm. This was found to retrieve the reported binding mode of **(III)** in the X-ray crystal structure of the active site of VEGFR-2, with root mean square difference (RMSD) between the top docking pose and original crystallographic geometry of 0.3737, indicating the validity of the selected docking algorithm (Fig. 17).

**Figure 18:**
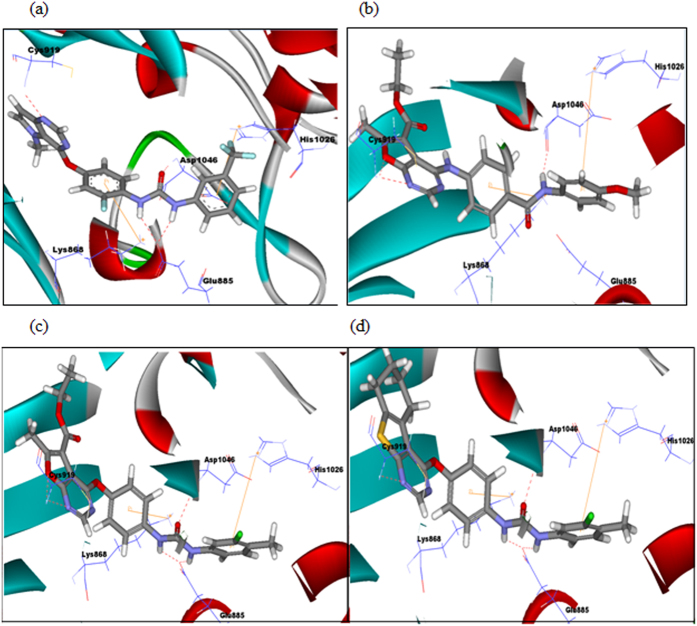
(**a)** Retrieved docking pose of the pyrimidine-based inhibitor **(III)** (PDB code 3VHE) showing the same key interactions as reported. **(b–d)** Docking poses of the target compounds *(**14b, 16e, 21e**)* to the ATP-binding pocket of VEGFR-2 in its inactive conformation. Compound ***(14b)*** missed one key interaction with with Glu885 residue, while compounds ***(16e, 21e)*** established the same key interactions as the lead compound.

Docking of the target compounds revealed that the core scaffolds adopted volumes and orientations in the hinge region as that of the lead compound. Compounds with urea linkage **(15a, 15b, 15c, 15f, 15g, 15i, 16a–e, 20a–g, 21a–e)** were shown to form the essential key interactions, known for type II VEGFR-2 inhibitors. Thus, a hydrogen bond was observed between the N1-nitrogen of the furo[2,3-*d*]pyrimidine or thieno[2,3-*d*]purimidine core, and the main chain NH group of Cys919 in the hinge region. The biarylurea tail was shown to extend towards the back hydrophobic pocket forming two hydrogen bonds; the two NH formed bifurcate hydrogen bonds with Glu885 while the urea carbonyl formed hydrogen bond with Asp1046. The substituted phenyl ring occupied the deep extended hydrophobic pocket created by the movement of Phe1047 of the ‘DFG’ motif to induce the ‘DFG-out’ conformation. Also, two Pi-cation interactions were revealed with Lys868 and His1026 residues (Fig. 18c,d). In addition, some of these compounds showed additional H-bond with Cys919 residue via the oxygen atom of furan ring **(15a, 15b, 15c, 15f, 15g, 15i, 16a–e)**, resulting in higher docking scores (Fig. 18c).

The network of interactions revealed by most of the urea-based derivatives may interpret their superior VEGFR-2 inhibitory activity as presented in the kinase assay.

On the other hand, the amide-based derivatives **(14a–e)**, missed one essential key interaction with Glu885 residue as an essential feature for type-II inhibitors (Fig. 18b). This interaction pattern was in line with their weaker activity observed in the *in vitro* kinase assay.

## Conclusion

Two series of pyrimidine-based derivatives namely the furo[2,3-*d*]pyrimidine and thieno[2,3-*d*]pyrimidine series, linked to either biarylamide or biarylurea via an NH or ether linker, were designed, synthesized and evaluated for their *in vitro* VEGFR-2 inhibitory activity as well as their anti-proliferative activity against NCI 60 cell line panel. Most of the biarylurea-based derivatives linked to either of the fused pyrimidine scaffolds exhibited good to potent VEGFR-2 inhibition at 10 μM concentration, with derivatives bearing an ether linkage generally exhibited better VEGFR-2 inhibition compared to their aniline analogues. Seven urea-based derivatives namely; The furo[2,3-*d*]pyrimidines **(15b, 16c, 16e)** and the thieno[2,3-*d*]pyrimidines **(21a, 21b, 21c, 21e)** exhibited potent dose-related VEGFR-2 inhibitory activity with IC_*50*_ values in nanomolar range. The thieno[2,3-*d*]pyrimidine derivative **(21e)** bearing 1-(3-chloro-4-methylphenyl)-3-phenyl urea via an ether linker at 4-position, exhibited a highly potent nanomolar inhibition of VEGFR-2 kinase (IC_*50*_ 21 nM). Results of further studies indicated that the most potent compounds **(16e, 21b, 21c, 21e)** showed good inhibitory activity against c-Kit and RET kinases in addition to VEGFR-2 kinase.

In addition, compounds **(15b)** (IC_*50*_ 946 nM), **(21b)** (IC_*50*_ 33.4 nM) and **(21c)** (IC_*50*_ 47 nM) manifested *in vitro* good to potent ability to inhibit VEGF-induced HUVEC cell line proliferation with inhibition percent of 99.5%, 81.97% and 79.15% respectively. In accordance with these *in vitro* findings, oral administration of compounds (**21b** and **21e)** at 5 and 10 mg/kg/day for 8 consecutive days demonstrated potent *in vivo* anticancer activity, eliciting apoptotic cell death in EAC solid tumor model. Besides, compounds (**21b** and **21e)** blunted angiogenesis in EAC solid tumor as evidenced by decreased VEGFR-2 phosphoryaltion and consequent reduction of %MVD. Miles vascular permeability assay also confirmed the antiangiogenic effects of **21b** and **21e** *in vivo*. Furthermore, such compounds increased active caspase 3 levels, hence, triggering apoptotic cell death in EAC solid tumor. Interestingly, such compounds did not show any noticeable toxicity.

These results were further explained using molecular docking studies which revealed the ability to the urea-based derivatives to form a network of key interactions, known to be essential for type II VEGFR-2 inhibitors. However, their amide-based analogues missed one key interaction with Glu885 residue.

## Experimental

### Chemistry and analysis

All used chemicals whether starting materials or reagents were purchased from Sigma-Aldrich (USA) or Alfa-Aesar Organics and used without further purification. Melting points were determined in one end open capillary tubes using Stuart Scientific apparatus and were uncorrected. The reactions were monitored using analytical thin layer chromatography (TLC) purchased from Merck (Merck, Darmstadt, Germany) and performed on on silica gel 60 packed on Aluminum sheets, with visualization under U.V. light (254 nm). The hydrogenation process was carried out using hydrogenator (Parr Shaker) apparatus. FT-IR spectra were recorded on a Perkin Elmer FT-IR spectrophotometer. ^1^HNMR spectra were run at Joel 300 MHz spectrophotometer and ^13^C NMR spectra were run at Joel 126 MHz spectrometer, in δ scale (ppm), *J* (Hz) using TMS as reference at Microanalytical Center at Cairo University. Mass spectra were recorded on Finnigan Mat SSQ 7000 (70 eV) mass spectrometer at Microanalytical Center at Cairo University. Elemental analyses were performed at Al-Azhar University at the regional center for mycology and biotechnology. Compounds (**1a–b**^23^− **2a**^43^**− 2b**^44^− **3a**^45^− **4**^26^− **5a**^46^− **5b,d,e**^47^− **5c**^48^**− 6a**^49^**− 6b,c**^50^**− 6e**^51^− **7a,b,c,e,h**^52^−**7i**^53^− **8a**^54^− **8e**^55^− **9a,b**^56^− **9d**^57^− **9h**^58^− **10**^30^− **17**^33^− **18**^34^− **19**^35^) were prepared according to the reported procedures.

#### N-(4-aminophenyl)-4-chlorobenzamide (3b)

To a solution of the 4-chloro nitro derivative **(2b)** (1g, 3.6 mmol) in ethanol (100 mL), Pd-C (0.1g, 10%) was added and then the mixture was stirred under H_2_ at room temperature, at 50 bar for 30 minutes. After removing the catalyst by filtration over celite, the filtrate was concentrated *in vacuo*, dried to afford the crystals of compound **(3b)** which were recrystallized from methanol. The titled compound was separated as white crystals (0.8 g, 80%); m.p. 178 °C; ^1^HNMR (300 MHz, DMSO-d_6_) δ 10.13 (s, 1H, NH D_2_O exchangeable), 7.97 (d, *J* = 9.0 Hz, 2H, ArH), 7.67 (d, *J* = 9.0 Hz, 2H, ArH), 7.39 (d, *J* = 8.7 Hz, 2H, ArH), 6.61 (d, *J* = 8.7 Hz, 2H, ArH), 5.53 (s, 2H, NH D_2_O exchangeable).

#### 4-Amino-N-(4-chloro-3-(trifluoromethyl)phenyl)benzamide (6d)

Compound (**6d**) was prepared through reduction of the respective nitro derivatives **(5d)** in a manner similar to that described for **3b**. The target compounds were recrystallized from ethyl acetate and hexane. The titled compound was separated as orange crystals (0.935 g, 85%); m.p. 152–155 °C; ^1^HNMR (300 MHz, DMSO-d_6_) δ 10.13 (s, 1H, NH D_2_O exchangeable), 8.36 (s, 1H, ArH), 8.10 (d, *J* = 9.0 Hz, 1H, ArH), 7.74 (d, *J* = 8.7 Hz, 2H, ArH), 7.64 (d, *J* = 9.0 Hz, 1H, ArH), 6.62 (d, *J* = 9.0 Hz, 2H, ArH), 5.53 (s, 2H, NH D_2_O exchangeable).

#### General procedure for the preparation of compounds (7a–i)

To a solution of *p*-nitroaniline (1 g, 6 mmol: 1 equiv.) in dry methylene chloride (20 mL), the appropriate isocyanate (6 mmol; 1 equiv.) was added and the mixture was stirred at room temprature for 24 hours. The formed solid was collected by filtration, stirred again with dry methylene chloride then filtered off and dried. Recrystallization was accomplished using ethanol giving compounds **(7a–i)** in yields (40–50%). (Details of **7a–i** are in supplementary data).

#### General procedure for the preparation of compounds (8a–i)

Compounds (**8a–i**) were prepared through reduction of the respective nitro derivatives **(7a–i)** in a manner similar to that described for **3a,b** while using methanol as the solvent. The target compounds were recrystallized from ethanol (Supplementary).

#### General procedure for the preparation of compounds (9a**–**e)

To a solution of *p*-aminophenol (1g, 7.5 mmol: 1 equiv.) in dry dioxane (10 mL), the appropriate isocyanate (7.5 mmol; 1 equiv.) was added and the mixture was stirred at room temprature for 1 hour. The formed solid was collected by filtration, washed with dioxane, allowed to dry and recrytallized from acetone giving compounds **(9a–e)** in yields (75–99.8%) (Supplementary).

#### Ethyl 6-methyl-4-oxo-3,4-dihydrofuro[2,3-d]pyrimidine-5-carboxylate (11)

Acetic anhydride (23 mL, 492.38 mmol: 23.8 equiv.) was added portionwise to stirred formic acid (46 mL, 614.92 mmol: 29.8 equiv.) at 0 °C and stirring was continued for 30 minutes, after which compound **(10)** (4 g, 20.6 mmol: 1 equiv.) was added, the ice bath was then removed. The mixture was heated under reflux at 130 °C for 48 hours. The solvent was evaporated under vaccum and the resultant solid was washed with diethyl ether, dried, recrystallized from hexane and ethyl acetate to afford buff crystals of the titled furo[2,3-*d*]pyrimidine **(11)** (3.9 g, 87%); m.p. 206–208 °C; ^1^HNMR (300 MHz, DMSO-d_6_) δ 12.56 (s, 1H, NH D_2_O exchangeable), 8.09 (s, 1H, pyrimidine H), 4.27 (q, *J* = 7.1 Hz, 2H, -CH_2_CH_3_), 2.68 (s, 3H, CH_3_), 1.30 (t, *J* = 7.1 Hz, 3H, -CH_2_CH_3_).

#### Ethyl 4-chloro-6-methylfuro[2,3-d]pyrimidine-5-carboxylate (12)

A mixture of the furo[2,3-*d*]pyrimidine **(11)** (3.5 g, 15.57 mmol: 1 equiv.) and phosphorous oxychloride (29 mL, 278 mmol: 18.2 equiv.) was heated under reflux for 3 hours. The mixture was then slowly poured on ice/water, then neutralized using ammonia solution (33%, 50 mL), then extracted with ethyl acetate (2*50 mL) .The combined organic layer was separated, dried over anhydrous Na_2_SO_4_ and the solvent was evaporated under vacuum and to afford brown oil that was solidified upon cooling to give the titled compound **(12)** as light brown crystals (2.7 g, 76%) which was used directly in the next reaction.

#### General procedure for the preparation of compounds (13a,b)

To a solution of the 4-chloro furo[2,3-*d*]pyrimidine derivative **(12)** (0.25 g, 1 mmol: 1 equiv.) in ethanol (15 mL), the respective N-(4-aminophenyl) benzamide derivative **(3a,b)** (1 mmol; 1 equiv.) and TEA (0.3 mL, 2 mmol; 2 eq.) were added. The mixture was heated under reflux for 18–24 hours. The resultant solid was collected by filtration, washed with hot ethanol, allowed to dry and recrystallized from THF to give the titled compounds **(13a,b)** in yields (67–70%) (Supplementary).

#### General procedure for the preparation of compounds (14a**–**e)

A solution of the 4-chloro furo[2,3-*d*]pyrimidine derivative **(12)** (0.25 g, 1 mmol: 1 equiv.), the appropriate 4-amino-N-phenyl benzamide derivative **(6a–e)** (1 mmol; 1 equiv.) and TEA (0.3 mL, 2 mmol; 2 eq.) in ethanol (15 mL) was heated under reflux for 24 hours (Supplementary).

#### General procedure for the preparation of compounds (15a**–**i)

Compounds (**15a–i**) were prepared from the 4-chloro furo[2,3-*d*]pyrimidine derivative **(12)** and the respective aminophenyl urea derivative **(8a–i)** in a manner similar to that described for (**13a–b**). The target compounds were recrystallized from acetone (Supplementary).

#### General procedure for the preparation of compounds (16a**–**e)

A solution of the respective hydroxyphenyl urea derivative **(9a–e)** (1 mmol: 1 equiv.) and cesium carbonate (2 mmol; 2 equiv.) in dry acetonitrile (10 mL) was stirred at room temperature for 1 hour, after which, the 4-chloro furo[2,3-*d*]pyrimidine derivative **(12)** (0.25 g, 1 mmol; 1 equiv.) was added and the mixture was heated at 55–60 °C for 3–4 hours. The solvent was evaporated under vaccum and the resultant solid was stirred with cold NaOH solution (1 M, 20 mL), filtered off, dried, recrystallized from acetonitrile to afford the target compounds **(16a–e)** in yields (51–80%) (Supplementary).

#### General procedure for the preparation of compounds (20a**–**g)

Compounds (**20a–g**) were prepared from the 4-chloro thieno[2,3-*d*]pyrimidine derivative **(19)** and the respective aminophenyl urea derivative **(8a–g)** in a manner similar to that described for (**13a–b**). The target compounds were recrystallized from acetone (Supplementary).

#### General procedure for the preparation of compounds (21a**–**e)

Compounds (**21a–e**) were prepared from the 4-chloro thieno[2,3-*d*]pyrimidine derivative **(19)** and the respective hydroxyl phenyl urea derivative **(9a–e)** in a manner similar to that described for (**16a–e**). The target compounds were recrystallized from acetonitrile (Supplementary).

### Biological Evaluation assay

#### In vitro VEGFR-2 tyrosine kinase activity

The *in vitro* enzyme inhibition determination for the synthesized compounds was carried out in BPS Bioscience Corporation, San Diego, CA, USA (www.bpsbioscience.com).

The VEGFR-2 tyrosine kinase activity at single dose concentration of 10 μM was performed, where VEGFR-2 (KDR) (BPS#40301) served as the enzyme source and Poly (Glu, Tyr) sodium salt, (4:1, Glu:Tyr) (Sigma#P7244) served as the standardized substrate & Kinase-Glo Plus Luminescence kinase assay kit (Promega#V3772) (Supplementary).

#### In vitro multiple kinases inhibition assay

The *in vitro* multiple kinases inhibition adetermination for the synthesized compounds was carried out in BPS Bioscience Corporation, San Diego, CA, USA (www.bpsbioscience.com).

The multiple kinases activity at single dose concentration of 10 μM was performed, c-Kit, c-Raf, c-Src, RET served as the enzyme source. Poly (Glu, Tyr) sodium salt, (4:1, Glu:Tyr) (Sigma#P7244) served as the standardized substrate for c-Kit, c-Scr kinases, while Inactive MEK1 (BPS Bioscience) and IGF-1Rtide (Anaspec) is the standardized substrate for c-Raf and RET kinases respectively. Kinase-Glo Plus Luminescence kinase assay kit (Promega#V3772), ADP-Glo Luminescence assay kit was used for c-kit assay (Supplementary).

#### In vitro HUVEC Anti-proliferative assay

The *In vitro* HUVEC proliferative assay for the synthesized compounds was also carried out in BPS Bioscience Corporation, San Diego, CA, USA (www.bpsbioscience.com).

The assay was performed at single dose concentration of 10 μM, where HUVEC umbilical vein endothelial cells, human (Life Technologies#C-003-5C) served as the cells’ source, in Medium 200 (Life Technologies#M-200-500), with large vessel endothelial supplement (LVES) (Life Technologies#A14608-01) and Pen-step (Hyclone#SV30010). AlamarBlue (Life Technologies#DAL1025) was used as the fluorescent reagent (Supplementary).

#### In vivo anticancer activity assessment

Animals. Female Swiss albino mice weighing 15–20 g were used in the present study. They were obtained from the animal breeding laboratory, National Cancer Institute (NCI), Cairo University, Egypt. Animals were kept in our animal facility, faculty of pharmacy, Ain Shams university at 25 ± 2 °C and a relative humidity of 40–45% with alternative day and night cycles of 12 h each. Animals had free access to pellet food and water *ad libitum*. Standard diet pellets (El-Nasr, Abu Zaabal, Egypt) contained not less than 20% protein, 5% fiber, 3.5% fat, 6.5% ash and a vitamin mixture. Animal care and experiments were conducted in accordance with the protocols approved by the Ethics Research Committee, Faculty of Pharmacy, Ain Shams University, following the Institutional Animal Care and Use Committee guidelines.

Tumor cells preparation and transplantation. Ehrlich ascites carcinoma (EAC) cells were obtained from NCI, Cairo University, Egypt. EAC cells were maintained in the ascitic form in Swiss albino mice by intraperitoneal transplantation of each mouse every 10 days. Ascitic fluid was drawn from tumor-bearing mice at the log phase (7–8^th^ day of tumor bearing) of the tumor cells. The freshly drawn fluid was diluted with ice-cold sterile isotonic saline. 200 μl tumor cell suspension containing 2 × 10^6^ tumor cells were then subcutaneously injected in each mouse.

Experimental design. Swiss albino mice were divided into six groups (n = 6 per group). After subcutaneous inoculation of EAC cells, mice developed palpable mass (tumor volume range (200–250 mm^3^) – day 0. Animals were then treated for 8 consecutive days as described below:

- Group I served as control (5% DMSO) (10 ml/kg)
- Groups II and III received compound 21b at 5 and 10 mg/kg orally respectively
- Groups IV and V received compound 21e at 5 and 10 mg/kg orally respectively
- Group VI was administered sorafenib p-toluenesulfonate (10 mg/kg po) (LC laboratories, Cat. No. S-8502)

Evaluation of effectiveness. Tumor growth curve was drawn according to the change in tumor volume from first day of treatment (day 0) till day 7. Longest and shortest diameters of the solid tumor were monitored using a digital vernier caliper. Tumor volume (TV) of each animal was calculated using the following formula:


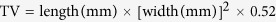


Tumor growth inhibition (TGI) was calculated as follows.

TGI(%) = 1 − (RTV of the treated group at the day of measurement)/(RTV of control group at the day of measurement) × 100. RTV = (Tumor volume at the day of measurement)/Tumor volume at the initial day.

On day 8, animals were sacrificed and tumor specimens were excised, weighed and fixed in neutral 10% buffered formalin (pH 7.2) for histopathological examination using light microscopy as well as immunohistochemistical detection of phospho-VEGFR-2 (Tyr951), CD31, CD34 and active caspase 3 protein levels (Supplementary).

Miles Vascular Permeability Assay. One to two days prior to the experiment, mice were shaved to expose the skin. Mice were anesthetized with intra-peritoneal injection of Avertin. A sterile solution of 1% Evans Blue (EB) in PBS was prepared and 100 μl was injected IV into the tail vein, using a needle (small gauge, 27–30) at a 10–15 degree angle. The animals were then left for (30–60) min. VEGF (50 μl of 1 ng/μl) and 50 μl of PBS with 0.05% gelatin were injected intradermally using a 30-gauge needle into the skin overlying the back. After 20 min, the animals were sacrificed by cervical dislocation that is recommended to limit significant interference with vascular permeability. The skin was opened, exposed and representative pictures were taken to assess the intensity of EB 500 μl formamide for 5 days. The Formamide/EB mixture was centrifuged and supernatant absorbance was measured at 600 nm (Shimadzu Spectrophotometer, UV-1601; Japan). extravasation was calculated as ng EB per mg tissue^59^.

#### Statistical analysis

Data are expressed as mean ± standard deviation (SD). Statistical analysis was carried out using one-way analysis of variance (ANOVA) followed by Dunnett or Tukey–Kramer as indicated as a post hoc test for multiple comparisons. The 0.05 level of probability was used as the criterion for significance. All statistical analyses were performed using Graphpad Instat software (version 2). Graphs were sketched using GraphPad Prism software (version 5).

### *In vitro* Anti-proliferative activity against 60 cell line panel

The NCI *in vitro* anticancer screening is a two-stage process, beginning with the evaluation of all compounds against the full NCI 60 cell lines panel representing leukemia, Non-Small Cell Lung Cancer, melanoma, colon cancer, CNS cancer, breast cancer, ovarian cancer, renal cancer and prostate cancer at a single dose of 10 μM. The output from the single dose screen is reported as a mean graph (Supplementary).

### Molecular docking study

The x-ray crystal structure of VEGFR-2 tyrosine kinase co-crystallized with pyrimidine-based compound **(III)** was obtained from the Protein Data Bank at the Research Collaboration for Structural Bioinformatics (RCSB) [www.rcsb.org] (PDB code: 3VHE) and loaded in Accelry’s discovery studio 2.5^19^. The protein structure was prepared using the default protein preparation tools integrated in the software. This was accomplished by adding hydrogen atoms to the amino acid residues, completing the missing residues and applying force field parameters by using CHARMm^60^ force field. Water molecules were preserved because of their importance in ligand interaction to VEGFR-2 enzyme. The protein structure was minimized using 500 steps employing SMART minimizer algorithm. Also binding pocket together with the surrounding amino acid residues was identified. The lead structure was removed from the binding sites. Our ligands were prepared using ligand preparation protocol of Accelry’s Discovery Studio. The ionization pH was adjusted to 7.4, hydrogen atoms were added and no isomers or tautomers were generated from the ligands. Docking was carried out using C-Docker software in the interface of Accelry’s discovery studio 2.5. The default values of C-Docker were used but with enabling early termination and allowing generating diverse solutions to get more possible docking solutions. Also, the number of docking iterations was raised to 500,000 ones so high number of predictions was obtained. The kinase scoring function, modified from the ChemScore Fitness Function (KSC), was applied in all docking calculations. Ten docking poses were generated for each ligand docked and were thoroughly inspected for getting the best binding mode^61^.

## Additional Information

**How to cite this article**: Aziz, M. A. *et al*. Discovery of Potent VEGFR-2 Inhibitors based on Furopyrimidine and Thienopyrimidne Scaffolds as Cancer Targeting Agents. *Sci. Rep*. **6**, 24460; doi: 10.1038/srep24460 (2016).

## Supplementary Material

Supplementary Information

## Figures and Tables

**Figure 10:**
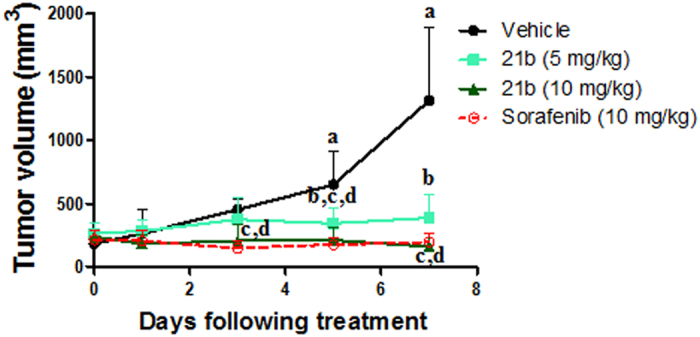
Effects of compound *(21b)* (5 and 10 mg/kg) or sorafenib p-toluenesulfonate treatment on tumor growth in EAC solid tumor bearing mice. Values are given as mean ± SD (n = 6). ^**a**^Statistically significant difference from control (day 0) at P < 0.05 using one way ANOVA followed by Dunnett as a post hoc test ^**b,c,d**^Statistically significant difference of **21b** (5 mg/kg), **21b** (10 mg/kg) and sorafenib respectively from the corresponding vehicle treated group at P < 0.05 using one way ANOVA followed by Tukey– Kramer as a post hoc test.

**Figure 11:**
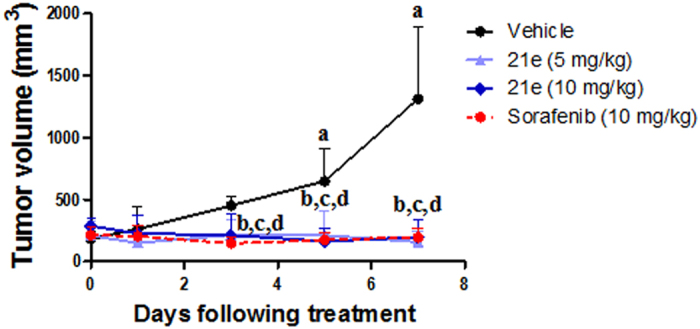
Effects of compound *(21e)* (5 and 10 mg/kg) or sorafenib p-toluenesulfonate treatment on tumor growth in EAC solid tumor bearing mice. Values are given as mean ± SD (n = 6). ^**a**^Statistically significant difference from control (day 0) at P < 0.05 using one way ANOVA followed by Dunnett as a post hoc test ^**b,c,d**^Statistically significant difference of **21e** (5 mg/kg), **21e** (10 mg/kg) and sorafenib respectively from the corresponding vehicle-treated group at P < 0.05 using one way ANOVA followed by Tukey–Kramer as a post hoc test.

**Figure 12:**
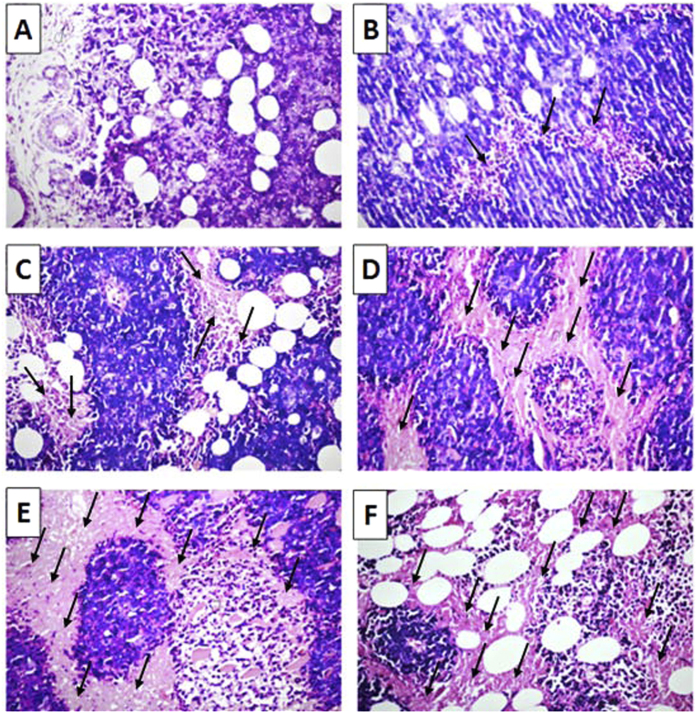
Histolopathological examination of hematoxylin-eosin sections of mice EAC tumor (40x). (**A**) Section taken from the control group shows intact tumor cells in the subcutaneous tissue and between the skeletal muscles. (**B,C)** Sections taken from the groups treated with **21b** at 5 mg/kg (**B**) & 10 mg/kg (**C**) showing mild focal necrosis (**B**) and moderate diffuse necrosis (**C**) as indicated by arrows. (**D,E)** Sections taken from the groups treated with **21e** at 5 mg/kg (**D**) & 10 mg/kg (**E**) showing moderate diffuse necrosis (**D**) and severe necrosis in a wide area (**E**) as indicated by arrows. (**F)** Section taken from the group treated with Sorafenib (10 mg/kg) showing wide severe necrosis as indicated by arrows.

**Figure 13:**
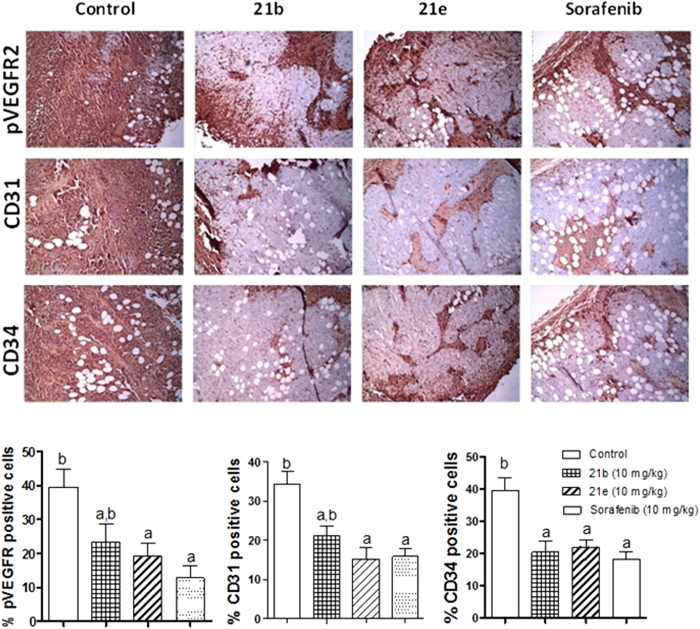
Effects of compounds *(21b, 21e)* on pVEGFR-2, CD31 and CD34 expressions in Ehrlich ascites carcinoma (EAC) solid tumor. (**A**) Immunohistochemical staining of pVEGFR-2, CD31 and CD34 in EAC solid tumor sections (100x): vehicle, 21b (10 mg/kg), 21e (10 mg/kg) and sorafenib (10 mg/kg) treated EAC-bearing mice. (**B)** Quantification of pVEGFR-2, CD31 and CD34 staining was calculated as area percentage of immunopositive cells to the total area of the microscopic field across seven fields. Values are given as mean ± SD. a and b: Statistically significant difference from vehicle and sorafenib-treated groups respectively at P < 0.05 using one way ANOVA followed by Tukey–Kramer as a post *hoc* test.

**Figure 14:**
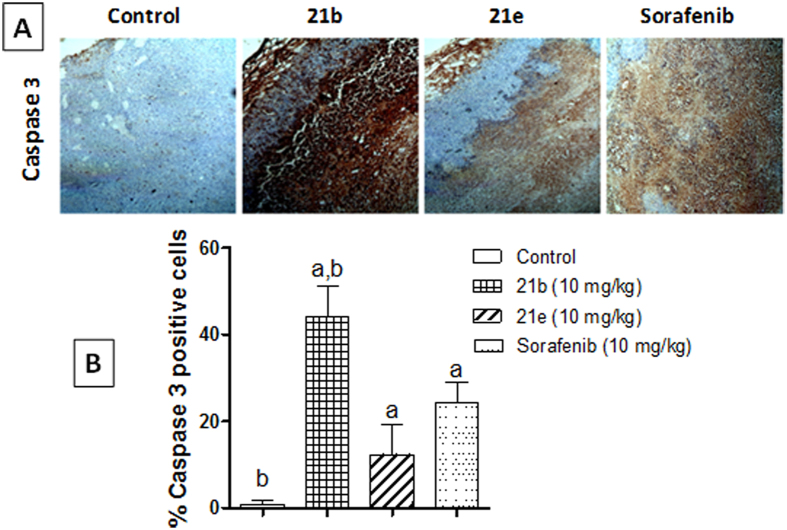
Effects of compounds *(21b, 21e)* on caspase 3 expression in Ehrlich ascites carcinoma (EAC) solid tumor. (**A**) Immunohistochemical staining of caspase 3 in EAC solid tumor sections (100x): vehicle, **21b** (10 mg/kg), **21e** (10 mg/kg) and sorafenib (10 mg/kg) treated EAC-bearing mice. (**B)** Quantification of caspase 3 staining was calculated as area percentage of immunopositive cells to the total area of the microscopic field across seven fields. Values are given as mean ± SD. (a and b: Statistically significant difference from vehicle and sorafenib-treated groups respectively at P < 0.05 using one way ANOVA followed by Tukey–Kramer as a post *hoc* test.

**Figure 15:**
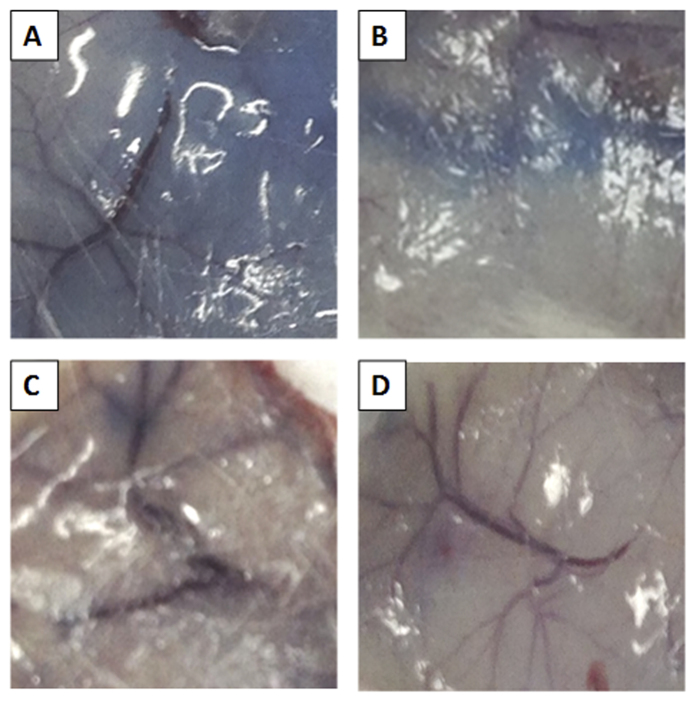
Representative pictures of skin tissues showing EB Extravasation. (**A)** the control group. (**B)** the group treated with ***21b*** (10 mg/kg), (**C)** the group treated with ***21e*** (10 mg/kg), (**D)** the group treated with Sorafenib (10 mg/kg).

**Figure 16:**
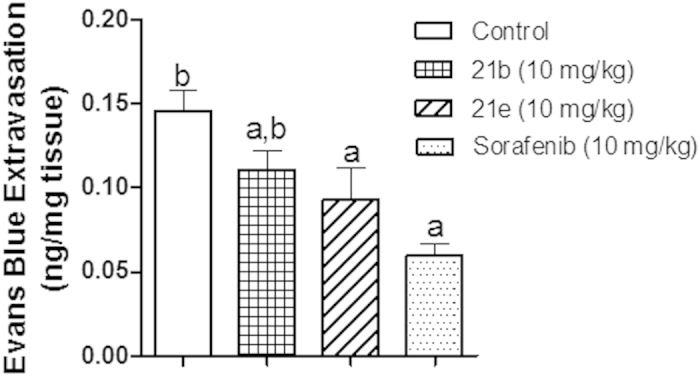
Quantitation of EB extravasation in skin tissues after incubation with 500 μl formamide to extract extravasated EB. Optical density was measured at 610 nm and the measurements converted into ng dye extravasated per mg tissue. Data are represented as mean ± SD, n = 6, Statistical analysis was performed using one-way ANOVA followed by Tukey-Kramar as a post-hoc test ^**a**^significantly different from the control group at p < 0.05 ^**b**^significantly different from the Sorafenib-treated group at p < 0.05.

**Table 1:** The IC_50_ values for the investigated compounds (*15b, 15f, 16b, 16c, 16e, 20g, 21a, 21b, 21c, 21e*).

Cpd ID	VEGFR-2 inhibition %	VEGFR-2 IC_*50*_	Cpd ID	VEGFR-2 inhibition %	VEGFR-2 IC_*50*_
15b	97	946 nM	20g	87	1.5 μM
15f	98	2.1 μM	21a	86	454 nM
16b	77	1.0 μM	21b	94	33.4 nM
16c	100	551 nM	21c	96	47.0 nM
16e	83	122 nM	21e	100	21 nM
			Sorafenib		90 nM

**Table 2:** Percent inhibition of multiple kinases enzymatic activity exhibited by the target compounds *(16e, 21b, 21c, 21e)* at 10 μM.

Cpd ID	Inhibition % at 10 μM				
	VEGFR-2	c-Kit	c-Raf	c-Src	RET
16e	83	72	53	1	84
21b	94	81	35	7	87
21c	96	83	26	8	91
21e	100	64	71	6	91
Staurosporine, 1 μM		93	N/A	98	96
Sorafenib, 1 μM		N/A	92	N/A	N/A

**Table 3:** The effect of Compounds (* 15b, 15f, 16b, 16c, 16e, 20g, 21a, 21b, 21c, 21e *) on HUVEC proliferation at 10 μM.

Cpd ID	% Cell growth	% Cell inhibition	Cpd ID	% Cell growth	% Cell inhibition
15b	0.50	99.50	20g	29.25	70.75
15f	97.68	2.32	21a	35.69	64.31
16b	73.35	26.65	21b	18.03	81.97
16c	32.11	67.86	21c	20.85	79.15
16e	88.89	11.11	21e	54.03	45.97

**Table 4:** Antitumor activities of compounds *(21b, 2le)* and sorafenib against EAC solid tumor model.

Groups	Dose (mg/kg/day), po	TGI (%)	Tumor Index
Control	--------	0	0.057^b^ ± 0.15
21b	5	70.7	0.041 ± 0.016
21b	10	86.4	0.035^a^ ± 0.015
21e	5	79.1	0.041 ± 0.007
21e	10	82.7	0.034^a^ ± 0.005
Sorafenib p-toluenesulfonate	10	80.8	0.023^a^ ± 0.007

Compounds were orally administered to mice once daily for 8 consecutive days at the indicated doses. TGI means tumor growth inhibition ratio.

Tumor Indices Data are presented as mean ± SD, n = 6, Statistical analysis was carried out by one way ANOVA, followed by Tukey-Kramar post-hoc test.

^a^statistical significance compared to the corresponding control group at p < 0.05.

^b^statistical significance compared to Sorafenib-treated group at p < 0.05.

**Table 5:** Effect of compounds *(21b, 2le)* and sorafenib on vital organs’ indices of mice with EAC solid tumor.

Groups	Dose (mg/kg/day), po	Liver Index	Kidney Index	Heart Index	Brain Index
Control	--------	0.056 ± 0.01	0.011 ± 0.001	0.0049 ± 0.0009	0.018 ± 0.002
21b	5	0.049 ± 0.01	0.012 ± 0.003	0.0048 ± 0.0014	0.0164 ± 0.004
21b	10	0.051 ± 0.017	0.011 ± 0.003	0.0046 ± 0.0017	0.0167 ± 0.004
21e	5	0.048 ± 0.006	0.012 ± 0.002	0.0044 ± 0.0013	0.0167 ± 0.002
21e	10	0.057 ± 0.007	0.013 ± 0.002	0.0056 ± 0.0009	0.0174 ± 0.002
Sorafenib p-toluenesulfonate	10	0.052 ± 0.005	0.013 ± 0.003	0.0057 ± 0.0009	0.018 ± 0.002

Data are presented as mean ± SD (n = 6).
