# Supplementary material for: Discovery of Potent VEGFR-2 Inhibitors based on Furopyrimidine and Thienopyrimidne Scaffolds as Cancer Targeting Agents
Source: Sci Rep. 2016 Apr 15;6:24460. doi: 10.1038/srep24460 (PMC4832243; doi:10.1038/srep24460)
Supplement: Supplementary Information [file srep24460-s1.pdf]

Discovery of Potent VEGFR-2 Inhibitors based on Furopyrimidine and Thienopyrimidne Scaffolds as Cancer Targeting Agents

Marwa A. Aziz [a], Rabah A.T. Serya [a], Deena S. Lasheen [a]* Amal Kamal Abdel-Aziz [b], Ahmed Esmat [b], Ahmed M. Mansour [c]*, Abdel Nasser B. Singab [d]* and Khaled A.M. Abouzid [a]*

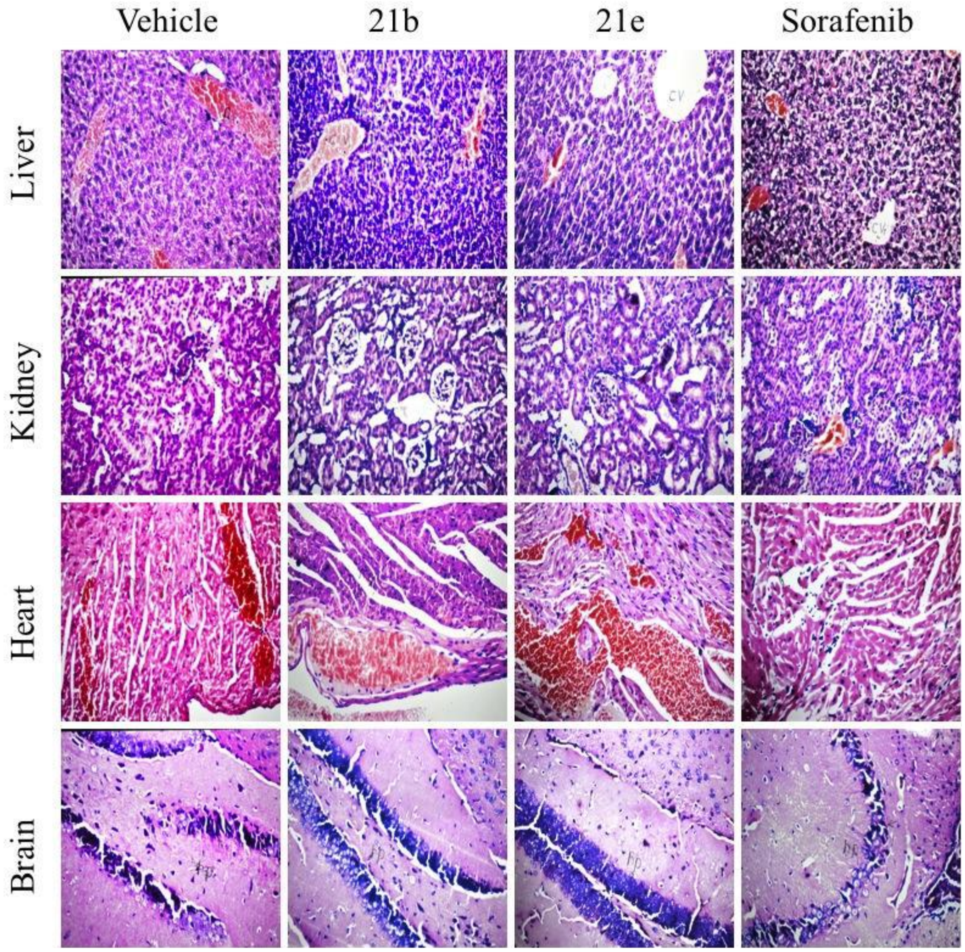

Figure S1. Histopathological examination of liver, kidney, heart and brain samples of vehicle, **21b** (10 mg/kg), **21e** (10 mg/kg) and sorafenib (10 mg/kg)-treated EAC solid tumor bearing mice (40x). Vehicle and 10 mg/kg **21b** treated EAC solid tumor bearing mice showed congested central hepatic vein and focally degenerated hepatocytes alongside with normal renal histoarchitecture. Congested myocardial blood vessels and neuronal degeneration were also noticed in hippocampus. 10 mg/kg **21e** treated group also showed congested myocardial blood vessels without any histopathological alterations in liver, kidney and brain specimens of treated mice. Degenerative changes were found in the hepatocytes and hippocampus of 10 mg/kg sorafenib treated EAC solid tumor bearing mice together with normal renal and cardiac histoarchitecture.

Table S1. Cell growth percentage of NCI 60 cancer cell lines exhibited by final compounds ***(14a, 14b , 14e, 15d, 15i, 20a, 20c, 20d, 21a, 21b, 21c, 21d):***

Panel/ Cell line	*Cell Growth Percent for the tested compounds*											
	14a	***14b***	***14e***	***15d***	***15i***	***20a***	***20c***	***20d***	***21a***	***21b***	***21c***	***21d***
Leukemia												
**CCRF-CEM**	93.8	89.1	80.9	**31.5**	93.7	92.3	94.6	97.7	64.9	70.0	64.4	77.9
**HL-60(TB)**	104.7	88.6	87.6	88.4	102.9	97.0	91.1	98.9	101.7	98.4	105.8	100.5
**K-562**	87.0	106.6	88.9	67.7	98.0	99.7	97.2	110.0	**26.1**	57.9	64.6	84.2
**MOLT-4**	94.0	92.3	105.0	**49.2**	98.6	89.4	81.3	92.3	54.0	58.1	**46.9**	72.0
**RPMI-8226**	94.6	91.3	84.5	**46.1**	95.0	99.2	102.1	106.3	**50.5**	58.9	**46.7**	68.8
**SR**	74.8	74.6	89.2	53.6	81.6	97.8	84.6	101.6	**24.3**	55.8	**50.9**	72.4
Non-Small Cell Lung Cancer												
**A549/ATCC**	95.3	93.3	56.6	**32.3**	89.2	73.8	78.3	74.2	67.8	66.5	61.2	66.6
**EKVX**	102.1	91.1	90.3	72.3	101.9	102.1	89.6	96.9	60.8	62.8	66.2	82.9
**HOP-92**	92.0	78.8	61.5	**50.6**	94.3	100.5	118.3	102.3	78.4	80.9	79.0	102.0
**NCI-H226**	97.0	94.2	63.1	54.8	104.0	89.8	90.1	102.7	66.6	73.1	65.1	74.0
**NCI-H23**	100.1	95.6	81.8	64.0	90.7	100.0	103.7	103.3	66.4	72.4	67.7	87.4
**NCI-H322M**	99.7	111.9	92.9	83.5	101.5	105.5	98.8	103.8	99.7	91.2	95.8	103.4
**NCI-H460**	100.0	97.0	82.9	67.1	106.4	103.5	106.	109.8	70.7	65.2	65.7	82.1
**NCI-H522**	78.0	84.0	66.9	57.1	83.7	92.9	81.16	92.10	**15.6**	**16.1**	**21.0**	**47.5**
Colon Cancer												
**HCC-2998**	105.2	113.6	91.4	104.8	114.0	95.3	117.5	103.2	89.6	104.1	101.7	107.1
**HCT-116**	85.5	85.1	64.7	50.7	78.7	114.5	90.99	112.3	67.4	62.1	55.8	82.6
**HCT-15**	107.5	88.5	92.2	58.9	105.2	110.3	97.8	104.2	**49.2**	74.9	67.1	91.6
**HT29**	83.6	81.1	58.8	**37.1**	103.1	96.1	103.9	87.8	74.3	65.6	54.8	86.4
**KM12**	93.8	96.1	89.0	61.0	103.1	102.1	89.6	104.7	**46.4**	**37.3**	**27.5**	69.1
**SW-620**	106.4	111.7	104.6	88.6	112.8	106.0	111.5	108.2	92.0	103.2	96.3	97.7
CNS Cancer												
**SF-268**	103.4	101.	74.1	86.4	103.0	100.0	99.5	105.9	76.3	72.6	72.0	87.0
**SF-295**	115.1	99.3	90.4	79.5	108.9	102.3	99.0	96.8	64.9	67.6	76.1	100.6
**SF-539**	107.2	63.8	58.9	**16.6**	89.	104.0	60.2	90.8	**44.0**	59.3	53.0	70.9
**SNB-19**	102.0	107.6	84.0	84.6	97.9	95.93	95.22	108.4	63.1	72.0	70.2	79.5
**SNB-75**	105.4	87.2	**7.0**	70.6	80.5	93.3	62.7	94.4	**45.6**	51.9	**50.3**	70.8
Melanoma												
**LOX IMVI**	93.1	93.2	75.2	64.1	100.3	99.4	94.4	103.0	69.5	77.3	61.5	84.8
**MALME-3M**	121.5	120.1	89.5	81.4	100.6	101.9	79.9	97.9	84.2	103.5	88.5	98.9
**M14**	95.2	100.6	81.2	**48.6**	99.9	102.8	89.8	99.3	56.5	68.2	67.3	87.0
**MDA-MB-435**	106.0	106.0	87.5	65.8	101.8	111.8	98.5	104.1	**19.2**	79.3	73.1	93.7
**SK-MEL-2**	99.78	107.3	82.4	72.7	119.7	113.7	116.0	115.9	87.6	99.7	97.3	104.1
**SK-MEL-28**	104.6	95.2	87.8	61.8	104.9	116.3	93.3	104.1	86.6	91.6	83.7	92.6
**SK-MEL-5**	92.2	87.9	85.6	52.9	93.7	97.3	92.3	98.1	67.9	72.9	63.6	78.4

Panel/ Cell line	Cell Growth Percent for the tested compounds											
	14a	*14b*	*14e*	*15d*	*15i*	*20a*	*20c*	*20d*	*21a*	*21b*	*21c*	*21d*
**UACC-257**	116.9	94.7	79.6	83.9	111.4	93.5	94.9	93.8	90.4	107.3	100.1	91.4
**UACC-62**	67.6	93.4	94.9	69.1	108.1	97.8	106.5	106.5	66.5	75.7	70.9	73.2
Ovarian Cancer												
**IGROV1**	104.8	108.2	101.8	78.4	109.5	107.0	105.8	113.0	87.2	89.0	88.7	107.8
**OVCAR-3**	109.5	109.5	97.2	80.8	117.9	103.2	99.1	109.6	74.9	63.5	68.3	85.8
**OVCAR-4**	98.0	95.6	**45.5**	73.7	104.9	107.1	88.8	100.9	55.1	61.2	56.6	93.6
**OVCAR-5**	115.7	114.0	109.9	121.8	107.4	119.2	106.5	107.7	106.8	113.7	104.7	113.3
**OVCAR-8**	102.9	98.9	76.6	**50.2**	112.2	107.1	112.9	111.8	93.5	85.9	89.9	94.7
**NCI/ADR-RES**	99.40	88.7	76.9	**43.7**	97.2	106.7	106.0	112.6	63.2	71.5	70.5	85.0
**SK-OV-3**	112.0	94.1	85.5	93.2	88.2	99.5	88.4	98.6	72.2	79.7	78.2	90.8
Renal Cancer												
**786-0**	96.3	92.1	74.9	79.1	99.8	109.5	100.	102.7	84.8	87.0	97.6	108.2
**A498**	92.4	75.3	77.5	63.0	108.6	83.3	91.3	86.7	56.7	68.3	67.8	70.1
**ACHN**	113.6	95.2	88.7	57.4	98.8	101.4	92.0	99.7	78.7	73.4	75.0	95.4
**CAKI-1**	99.2	96.6	92.5	90.8	98.3	100.2	97.0	97.2	83.4	86.0	80.8	95.4
**RXF 393**	76.8	92.0	71.7	63.6	98.2	101.7	115.7	115.0	72.0	79.0	74.0	87.7
**SN12C**	94.1	103.3	99.3	73.9	107.1	99.2	91.0	101.8	83.3	75.9	78.2	86.5
**TK-10**	101.9	101.6	102.0	79.1	111.6	124.4	129.0	107.6	86.1	72.5	73.5	88.3
**UO-31**	89.5	94.3	97.3	**50.3**	94.7	71.5	61.0	90.9	55.2	59.0	52.5	71.2
Prostate Cancer												
**PC-3**	78.3	83.6	59.2	**41.2**	90.7	89.8	89.6	96.0	**33.4**	**37.5**	**45.0**	57.6
**DU-145**	75.9	97.1	82.1	70.8	103.3	109.3	104.7	110.1	86.1	81.28	82.4	91.9
Breast Cancer												
**MCF7**	90.6	95.6	78.6	**43.3**	94.9	96.2	86.2	91.9	**44.5**	**44.1**	54.4	83.2
**MDA-MB-231/ATCC**	95.8	91.0	85.6	**18.6**	94.6	91.6	89.0	101.3	66.2	81.9	69.6	70.1
**HS 578T**	107.3	83.7	51.2	80.6	97.7	85.3	74.4	103.2	73.6	76.8	60.9	74.7
**BT-549**	87.6	61.9	67.3	61.5	84.4	108.7	85.8	100.0	63.8	71.9	88.5	112.5
**T-47D**	98.2	91.0	65.1	**1.9**	78.2	100.5	66.4	100.4	**37.9**	**49.2**	**41.7**	73.3
**MDA-MB-468**	90.9	102.0	90.7	49.7	128.2	96.8	114.3	109.9	53.6	87.2	69.3	102.6
**MEAN % Growth inhibition**	97.5	94.5	80.5	62.7	100.1	100.3	94.8	101.8	66.7	73.1	70.2	86.1

O 40-50% growth inhibition, **O** 50-60% growth inhibition, **O** 60-70% growth inhibition, **O** 70-80% growth inhibition, **O** 80-90% growth inhibition, **O** 90-100% growth inhibition

Experimental

Chemistry and analysis

1-(3-Chlorophenyl)-3-(4-nitrophenyl)urea (7d):

The titled compound was separated as white crystals (0.78 g, 42%); m.p. >295°C; ^{1}HNMR (300 MHz, DMSO-d_6) δ 9.52 (s, 1H, NH D_2O exchangeable), 9.04 (s, 1H, NH D_2O exchangeable), 8.32 (d, J = 8.2 Hz, 2H, ArH), 8.11 (s, 1H, ArH), 7.75 (d, J = 8.2 Hz, 2H, ArH), 7.63 (m, 2H, ArH), 7.00 (t, J = 8.0 Hz, 1H, ArH).

1-(3-Chloro-4-methylphenyl)-3-(4-nitrophenyl)urea (7f):

The titled compound was separated as white crystals (1.09 g, 50%); m.p. 182-185°C; ^{1}H NMR (300 MHz, DMSO-d_6) δ 9.45 (s, 1H, NH D_2O exchangeable), 9.20 (s, 1H, NH D_2O exchangeable), 8.22 (d, *J* = 8.9 Hz, 2H, ArH), 7.87 (d, *J* = 8.9 Hz, 2H, ArH), 7.66 (s, 1H, ArH), , 7.31 (d, *J* = 8.4 Hz, 1H, ArH), 7.21 (d, *J* = 8.4 Hz, 1H, ArH), 2.22 (s, 3H, Ph-CH_3).

1-(3-Acetylphenyl)-3-(4-nitrophenyl)urea (7g):

The titled compound was separated as white crystals (1 g, 50%); m.p. 297-300°C; ^{1}HNMR (300 MHz, DMSO-d_6) δ 9.55 (s, 1H, NH D_2O exchangeable), 9.21 (s, 1H, NH D_2O exchangeable), 8.20 (d, *J* = 8.7 Hz, 2H, ArH), 8.09 (s, 1H, ArH), 7.72 (d, *J* = 8.7 Hz, 2H, ArH), 7.63 (d, *J* = 8.1 Hz, 1H, ArH), 7.49-7.36 (m, 2H, ArH), 2.50 (s, 3H,-$COCH_3$).

1-(4-Aminophenyl)-3-(m-tolyl)urea (8b):

The titled compound was separated as greyish pink crystals (0.75 g, 75%); m.p. 183-187°C; ^{1}HNMR (300 MHz, DMSO-d_6) δ 8.39 (s, 1H, NH D_2O exchangeable), 8.11 (s, 1H, NH D_2O exchangeable), 7.25 (s, 1H, ArH), 7.19 (t, *J* = 7.9 Hz, 1H, ArH), 7.11 (d, *J* = 7.9 Hz, 1H, ArH), 7.05 (d, *J* = 8.7 Hz, 2H, ArH), 6.73 (d, *J* = 7.9 Hz, 1H, ArH), 6.15 (d, *J* = 8.7 Hz, 2H, ArH), 4.73 (s, 2H, NH D_2O exchangeable), 2.26 (s, 3H, Ph-CH_3).

1-(4-Aminophenyl)-3-(3-methoxyphenyl)urea (8c):

The titled compound was separated as white crystals (0.64 g, 72%); m.p. 164-168°C; ^{1}HNMR (300 MHz, DMSO-d_6) δ 8.52 (s, 1H, NH D_2O exchangeable), 8.15 (s, 1H, NH D_2O exchangeable), 7.16-7.10 (m, 2H, ArH), 7.05 (d, *J* = 8.0 Hz, 2H, ArH), 6.88 (d, *J* = 7.8 Hz, 1H, ArH), 6.51 (s, 1H, ArH), 6.49 (d, *J* = 8.0 Hz, 2H, ArH), 4.74 (s, 2H, NH D_2O exchangeable), 3.71 (s, 3H, OCH_3).

1-(4-Aminophenyl)-3-(3-chlorophenyl)urea (8d):

The titled compound was separated as buff crystals (0.68 g, 76%); m.p. 196-200°C; ^{1}HNMR (300 MHz, DMSO-d_6) δ 8.70 (s, 1H, NH D_2O exchangeable), 8.22 (s, 1H, NH D_2O exchangeable), 7.72 (s, 1H, ArH), 7.33 (d, *J* = 8.9 Hz, 1H, ArH), 7.22 (t, *J* = 8.9 Hz, 1H, ArH), 7.08 (d, *J* = 8.7 Hz, 2H, ArH), 6.94 (d, *J* = 8.9 Hz, 1H, ArH), 6.51 (d, *J* = 8.7 Hz, 2H, ArH), 4.77 (s, 2H, NH D_2O exchangeable).

1-(4-Aminophenyl)-3-(3-chloro-4-methylphenyl)urea (8f):

The titled compound was separated as grey crystals (0.73 g, 84%); m.p. 200-205°C; ^{1}HNMR (300 MHz, DMSO-d_6) δ 8.58 (s, 1H, NH D_2O exchangeable), 8.16 (s, 1H, NH D_2O exchangeable), 7.66 (s, 1H, ArH), 7.31 (d, *J* = 8.4 Hz, 1H, ArH), 7.21 (d, *J* = 8.4 Hz, 1H, ArH), 7.11 (d, *J* = 8.1 Hz, 2H, ArH), 6.52 (d, *J* = 8.1 Hz, 2H, ArH), 5.00 (s, 2H, NH D_2O exchangeable), 2.22 (s, 3H, Ph-CH_3).

1-(3-Acetylphenyl)-3-(4-aminophenyl)urea (8g):

The titled compound was separated as white crystals (0.675 g, 75%); m.p. 188-191°C; ^{1}HNMR (300 MHz, DMSO-d_6) δ 8.52 (s, 1H, NH D_2O exchangeable), 8.21 (s, 1H, NH D_2O exchangeable), 8.11 (s, 1H, ArH), 7.63 (d, *J* = 8.4 Hz, 1H, ArH), 7.50 - 7.33 (m, 2H, ArH), 7.15 (d, *J* = 8.1 Hz, 2H, ArH), 6.42 (d, *J* = 8.1 Hz, 2H, ArH), 4.79 (s, 2H, NH D_2O exchangeable), 2.25 (s, 3H, -$COCH_3$).

1-(4-Aminophenyl)-3-(4-ethylphenyl)urea (8i):

The titled compound was separated as white crystals (0.75 g, 75%); m.p. >300°C; ^{1}HNMR (300 MHz, DMSO-d_6) δ 8.29 (s, 1H, NH D_2O exchangeable), 8.06 (s, 1H, NH D_2O exchangeable), 7.31 (d, *J* = 8.7 Hz, 2H, ArH), 7.06 (d, *J* = 9.0 Hz, 2H, ArH), 6.83 (d, *J* = 8.7 Hz, 2H, ArH), 6.51 (d, *J* = 9.0 Hz, 2H, ArH), 4.17 (s, 2H, NH D_2O exchangeable), 3.97 (q, *J* = 6.9 Hz, 2H, Ph-$\underline{CH_2}CH_3$), 1.30 (t, *J* = 6.9 Hz, 3H, Ph-$CH_2\underline{CH_3}$).

1-(4-Hydroxyphenyl)-3-(3-methoxyphenyl)urea (9c):

The titled compound was separated as brown crystals (1.39 g, 85%); m.p. 221-224°C; ^{1}HNMR (300 MHz, DMSO-d_6) δ 9.03 (s, 1H, NH D_2O exchangeable), 8.51 (s, 1H, NH D_2O exchangeable), 8.28 (s, 1H, OH D_2O exchangeable), 7.22 (t, *J* = 7.8 Hz, 1H, ArH), 7.15 (d, *J* = 9 Hz, 2H, ArH), 6.89 (d, *J* = 7.8 Hz, 1H, ArH), 6.69 (d, *J* = 9 Hz, 2H, ArH), 6.66 (s, 1H, ArH), 6.51 (d, *J* = 7.8 Hz, 1H, ArH), 3.72 (s, 3H, OCH_3).

1-(3-Chloro-4-methylphenyl)-3-(4-hydroxyphenyl)urea (9e):

The titled compound was separated as brown crystals (1.39 g, 85%); m.p. 215-219°C; ^{1}HNMR (300 MHz, DMSO-d_6) δ 9.04 (s, 1H, NH D_2O exchangeable), 8.59 (s, 1H, NH D_2O exchangeable), 8.32 (s, 1H, OH D_2O exchangeable), 7.66 (s, 1H, ArH), 7.22 (d, *J* = 8.7 Hz, 2H, ArH), 7.16 (d, *J* = 8.1 Hz, 1H, ArH), 7.13 (d, *J* = 8.1 Hz, 1H, ArH), 6.71 (d, *J* = 8.7 Hz, 2H, ArH), 2.25 (s, 3H, Ph-CH_3).

Ethyl 4-[(4-(3-chlorobenzamido)phenyl)amino]-6-methylfuro[2,3-d]pyrimidine-5-carboxylate (13a):

The titled compound was separated as white crystals (0.335 g, 67%); m.p. 232-236°C; ^{1}HNMR (300 MHz, DMSO-d_6) δ 10.54 (s, 1H, NH D_2O exchangeable), 10.37 (s, 1H, NH D_2O exchangeable), 8.46 (s, 1H, pyrimidine H), 8.02 (s, 1H, ArH), 7.93 (d, *J* = 7.9 Hz, 1H, ArH), 7.78 (t, *J* = 7.9 Hz, 1H, ArH), 7.65 (d, *J* = 7.9 Hz, 1H, ArH), 7.59 (d, *J* = 8.0 Hz, 2H, ArH), 7.54 (d, *J* = 8.0 Hz, 2H, ArH), 4.46 (q, *J* = 7.1 Hz, 2H, -$\underline{CH_2}CH_3$), 2.75 (s, 3H, CH_3), 1.39 (t, *J* = 7.1 Hz, 3H, -$CH_2\underline{CH_3}$); ^{13}C NMR (126 MHz, DMSO-d_6) δ 165.12, 164.54, 163.57, 159.96, 153.73, 136.76, 134.80, 134.17, 133.13, 131.19, 130.23, 127.35, 126.40, 120.96, 119.72, 107.55, 10016, 61.98, 14.64, 13.81; FT-IR (ύ max, cm^{-1}): 3278 (NH), 3112 (CH aromatic), 2990 (CH aliphatic), 1695 (C=O amide), 1636 (C=N); MS : (Mwt. : 450) : *m/z* , 452 [M^{+}+2 , (7.9%)] , 450 [M^{+}, (30.6%)] , 139 (100%); Anal. Calcd for $C_{23}H_{19}ClN_4O_4$: C, 61.27; H, 4.25; N, 12.43; Found: C, 61.39; H, 4.28; N, 12.57.

Ethyl 4-[(4-(4-chlorobenzamido)phenyl)amino]-6-methylfuro[2,3-d]pyrimidine-5-carboxylate (13b):

The titled compound was separated as white crystals (0.35 g, 70%); m.p. >250°C; ^{1}HNMR (300 MHz, DMSO-d_6) δ 10.54 (s, 1H, NH D_2O exchangeable), 10.34 (s, 1H, NH D_2O exchangeable), 8.45 (s, 1H, pyrimidine H), 7.98 (d, *J* = 8.4 Hz, 2H, ArH), 7.77 (d, *J* = 8.4 Hz, 2H, ArH), 7.73 (d, *J* = 8.0 Hz, 2H, ArH), 7.61 (d, *J* = 8.0 Hz, 2H, ArH), 4.46 (q, *J* = 7.1 Hz, 2H, -$\underline{CH_2}CH_3$), 2.74 (s, 3H, CH_3), 1.39 (t, *J* = 7.1 Hz, 3H, -$CH_2\underline{CH_3}$); Anal. Calcd for $C_{23}H_{19}ClN_4O_4$ (Mwt. : 450): C, 61.27; H, 4.25; N, 12.43; Found: C, 61.37; H, 4.26; N, 12.57.

Ethyl 4-[(4-((3-chlorophenyl)carbamoyl)phenyl)amino]-6-methylfuro[2,3-d]pyrimidine-5-carboxylate (14a):

The titled compound was collected by filtration, washed with hot ethanol, allowed to dry and recrystallized from THF to give compound **(14a)** as white crystals (0.314 g, 67%); m.p. 226-230°C; ^{1}HNMR (300 MHz, DMSO-d_6) δ 10.73 (s, 1H, NH D_2O exchangeable), 10.24 (s, 1H, NH D_2O exchangeable), 8.46 (s, 1H, pyrimidine H), 7.97 (d, *J* = 8.7 Hz, 2H, ArH), 7.94 (s, 1H, ArH), 7.83 (d, *J* = 8.7 Hz, 2H, ArH), 7.67 (d, *J* = 9.0 Hz, 1H, ArH), 7.35 (t, *J* = 8.7 Hz, 1H, ArH), 7.13 (d, *J* = 9.0 Hz, 1H, ArH), 4.40 (q, *J* = 7.1 Hz, 2H, -$\underline{CH_2}CH_3$), 2.66 (s, 3H, CH_3), 1.36 (t, *J* = 7.1 Hz, 3H, -$CH_2\underline{CH_3}$);); ^{13}C NMR (126 MHz, DMSO-d_6) δ 164.91, 164.46, 164.39,

160.42, 153.36, 153.18, 141.96, 140.74, 132.84, 129.99, 128.57, 127.62. 122.91, 120.81, 119.54, 118.07, 107.20, 100.70, 62.01, 14.51, 13.63; FT-IR (ύ max, cm^{-1}): 3342 (NH), 3115 (CH aromatic), 2988 (CH aliphatic), 1683 (C=O amide), 1644 (C=N); MS : (Mwt. : 450) : *m/z* , 452 [M^++2 , (3.4%)] , 450 [M^+, (9.1%)] , 324 (100%); Anal. Calcd for $C_{23}H_{19}ClN_4O_4$: C, 61.27; H, 4.25; N, 12.43; Found: C, 61.39; H, 4.29; N, 12.58.

Ethyl 4-[(4-((4-methoxyphenyl)carbamoyl)phenyl)amino]-6-methylfuro[2,3-d]pyrimidine-5-carboxylate (14b):

The titled compound was collected by filtration, washed with hot ethanol, allowed to dry and recrytallized from THF to give compound **(14b)** as buff fine crystals (0.32 g, 70%); m.p. 218-222°C; ^{1}HNMR (300 MHz, DMSO-d_6) δ 10.93 (s, 1H, NH D_2O exchangeable), 8.52 (s, 1H, pyrimidine H), 7.95 (d, J = 8.7 Hz, 2H, ArH), 7.85 (d, J = 8.7 Hz, 2H, ArH), 7.56 (d, J = 9.0 Hz, 2H, ArH), 7.26 (s, 1H, NH D_2O exchangeable), 6.91 (d, J = 9.0 Hz, 2H, ArH), 4.47 (q, J = 7.1 Hz, 2H, -$\underline{CH_2}CH_3$), 3.81 (s, 3H, OCH_3), 2.76 (s, 3H, CH_3), 1.47 (t, J = 7.1 Hz, 3H, -$CH_2\underline{CH_3}$); FT-IR (ύ max, cm^{-1}): 3289 (NH), 3080 (CH aromatic), 2978 (CH aliphatic), 1681 (C=O amide), 1644 (C=N); Anal. Calcd for $C_{24}H_{22}N_4O_5$ (Mwt. : 446): C, 64.57; H, 4.97; N, 12.55; Found: C, 64.65; H, 5.01; N, 12.74.

Ethyl 6-methyl-4-[(4-(p-tolylcarbamoyl)phenyl)amino]furo[2,3-d]pyrimidine-5-carboxylate (14c):

The titled compound was collected by evaporating ethanol under vaccum and the resultant solid was stirred with ice/conc. HCl (10 mL), filtered off, allowed to dry and recrystallized from ethanol to give compound **(14c)** as brown crystals (0.3 g, 69%); m.p. 235-238°C; ^{1}HNMR (300 MHz, DMSO-d_6) δ 10.77 (s, 1H, NH D_2O exchangeable), 10.05 (s, 1H, NH D_2O exchangeable), 8.50 (s, 1H, pyrimidine H), 8.01 (d, *J* = 8.7 Hz, 2H, ArH), 7.91 (d, *J* = 8.7 Hz, 2H, ArH), 7.66 (d, *J* = 8.4 Hz, 2H, ArH), 7.14 (d, *J* = 8.4 Hz, 2H, ArH), 4.43 (q, *J* = 7.1 Hz, 2H, -$\underline{CH_2}CH_3$), 2.73 (s, 3H, CH_3), 2.38 (s, 3H, Ph-CH_3), 1.39 (t, *J* = 7.1 Hz, 3H, -$CH_2\underline{CH_3}$); FT-IR (ύ max, cm^{-1}): 3297 (NH), 3100 (CH aromatic), 2998 (CH aliphatic), 1685 (C=O amide), 1643 (C=N); Anal. Calcd for $C_{24}H_{22}N_4O_4$ (Mwt. : 430): C, 66.97; H, 5.15; N, 13.02; Found: C, 67.08; H, 5.18; N, 13.13.

Ethyl 4-[(4-((4-chloro-3-(trifluoromethyl)phenyl)carbamoyl)phenyl)amino]-6-methylfuro[2,3-d]pyrimidine-5-carboxylate (14d):

The titled compound was collected by filtration, washed with hot ethanol, allowed to dry and recrystallized from THF to give compound **(14d)** as yellowish white crystals (0.33 g, 60%); m.p. 238-240°C; ^{1}HNMR (300 MHz, DMSO-d_6) δ 10.82 (s, 1H, NH D_2O exchangeable), 10.52 (s, 1H, NH D_2O exchangeable), 8.55 (s, 1H, pyrimidine H), 8.37 (s, 1H, ArH), 8.11 (d, *J* = 8.9 Hz, 1H, ArH), 8.03 (d, *J* = 8.8 Hz, 2H, ArH), 7.94 (d, *J* = 8.8 Hz, 2H, ArH), 7.70 (d, *J* = 8.9 Hz, 1H, ArH), 4.46 (q, *J* = 7.1 Hz, 2H, -$\underline{CH_2}CH_3$), 2.75 (s, 3H, CH_3), 1.40 (t, *J* = 7.1 Hz, 3H, -$CH_2\underline{CH_3}$); FT-IR (ύ max, cm^{-1}): 3347 (NH), 3117 (CH aromatic), 2994 (CH aliphatic), 1682 (C=O amide), 1644 (C=N); Anal. Calcd for $C_{24}H_{18}ClF_3N_4O_4$ (Mwt. : 518): C, 55.55; H, 3.50; N, 10.80; Found: C, 55.62; H, 3.51; N, 10.89.

Ethyl 6-methyl-4-[(4-(phenethylcarbamoyl)phenyl)amino]furo[2,3-d]pyrimidine-5-carboxylate (14e):

The titled compound was collected by evaporating ethanol under vaccum and the resultant solid was stirred with ice/conc. HCl (10 mL), filtered again, allowed to dry and recrystallized from ethanol to give compound **(14e)** as white crystals (0.277 g, 60%); m.p. 176-180°C; ^{1}HNMR (300 MHz, DMSO-d_6) δ 10.89 (s, 1H, NH D_2O exchangeable), 8.51 (s, 1H, pyrimidine H), 7.91 (d, *J* = 8.4 Hz, 2H, ArH), 7.71 (d, *J* = 8.4 Hz, 2H, ArH), 7.31 (m, 5H, ArH), 6.17 (s, 1H, NH D_2O exchangeable), 4.47 (q, *J* = 7.1 Hz, 2H, -$\underline{CH_2}CH_3$), 3.72 (t, 2H, *J* = 6.6 Hz, -$\underline{CH_2}CH_2$-Ph), 2.96 (t, 2H, *J* = 6.6 Hz, -$CH_2\underline{CH_2}$-Ph), 2.76 (s, 3H, CH_3), 1.46 (t, *J* = 7.1 Hz, 3H, -$CH_2\underline{CH_3}$); FT-IR (ύ max, cm^{-1}): 3340 (NH), 3109 (CH aromatic), 2986 (CH aliphatic), 1685 (C=O amide), 1632 (C=N); Anal. Calcd for $C_{25}H_{24}N_4O_4$ (Mwt. : 444): C, 67.55; H, 5.44; N, 12.60; Found: C, 67.74; H, 5.51; N, 12.69.

Ethyl 6-methyl-4-[(4-(3-phenylureido)phenyl)amino]furo[2,3-d]pyrimidine-5-carboxylate (15a):

The titled compound was separated as white crystals (0.26 g, 60%); m.p. 281-283°C; ^{1}HNMR (300 MHz, DMSO-d_6) δ 10.40 (s, 1H, NH D_2O exchangeable), 8.60 (s, 1H, NH D_2O exchangeable), 8.58 (s, 1H, NH D_2O exchangeable), 8.41 (s, 1H, pyrimidine H), 7.68 (d, *J* = 9.0 Hz, 2H, ArH), 7.46 (d, *J* = 9.0 Hz, 2H, ArH), 7.44 (d, *J* = 8.1 Hz, 2H, ArH), 7.27 (t, *J* = 8.1 Hz, 2H, ArH), 6.96 (t, *J* = 8.1 Hz, 1H, ArH), 4.45 (q, *J* = 7.0 Hz, 2H, -$\underline{CH_2}CH_3$), 2.75 (s, 3H, CH_3),

1.40 (t, J = 7.0 Hz, 3H, -$CH_2\underline{CH_3}$); FT-IR (ύ max, cm^{-1}): 3299 (NH), 3040 (CH aromatic), 2980 (CH aliphatic), 1683 (C=O amide), 1638 (C=N); Anal. Calcd for $C_{23}H_{21}N_5O_4$ (Mwt. : 431): C, 64.03; H, 4.91; N, 16.23; Found: C, 64.18; H, 4.97; N, 16.37.

Ethyl 6-methyl-4-[(4-(3-(m-tolyl)ureido)phenyl)amino]furo[2,3-d]pyrimidine-5-carboxylate (15b):

The titled compound was separated as white crystals (0.207 g, 45%); m.p. >250°C; ^{1}HNMR (300 MHz, DMSO-d_6) δ 10.42 (s, 1H, NH D_2O exchangeable), 8.65 (s, 1H, NH D_2O exchangeable), 8.53 (s, 1H, NH D_2O exchangeable), 8.42 (s, 1H, pyrimidine H), 7.67 (d, J = 8.5 Hz, 2H, ArH), 7.47 (d, J = 8.5 Hz, 2H, ArH), 7.30 (s, 1H, ArH), 7.23 (d, J = 7.9 Hz, 1H, ArH), 7.15 (t, J = 7.9 Hz, 1H, ArH), 6.78 (d, J = 7.9 Hz, 1H, ArH), 4.45 (q, J = 7.1 Hz, 2H, -$\underline{CH_2}CH_3$), 2.74 (s, 3H, CH_3), 2.28 (s, 3H, Ph-CH_3), 1.40 (t, J = 7.1 Hz, 3H, -$CH_2\underline{CH_3}$); ^{13}C NMR (126 MHz, DMSO-d_6) δ165.72, 160.45, 154.47, 154.36, 153.01, 140.17, 138.40, 135.77, 133.55, 129.07, 122.94, 121.01, 119.39, 119.10, 115.77. 108.18, 100.57, 62.52, 21.70, 15.20, 14.39; FT-IR (ύ max, cm^{-1}): 3295 (NH), 3118 (CH aromatic), 2981 (CH aliphatic), 1660 (C=O amide), 1633 (C=N); Anal. Calcd for $C_{24}H_{23}N_5O_4$ (Mwt. : 445): C, 64.71; H, 5.20; N, 15.72; Found: C, 64.88; H, 5.27; N, 15.91.

Ethyl 4-[(4-(3-(3-methoxyphenyl)ureido)phenyl)amino]-6-methylfuro[2,3-d]pyrimidine-5-carboxylate (15c):

The titled compound was separated as white crystals (0.315 g, 65%); m.p. 220-222°C; ^{1}HNMR (300 MHz, DMSO-d_6) δ 10.41 (s, 1H, NH D_2O exchangeable), 8.65 (s, 2H, NH D_2O exchangeable), 8.41 (s, 1H, pyrimidine H), 7.67 (d, J = 8.0 Hz, 2H, ArH), 7.46 (d, J = 8.0 Hz, 2H, ArH), 7.37 (s, 1H, ArH), 7.17 (t, J = 8.4 Hz, 1H, ArH), 6.93 (d, J = 8.4 Hz, 1H, ArH), 6.54 (d, J = 8.4 Hz, 1H, ArH), 4.31 (q, J = 7.1 Hz, 2H, -$\underline{CH_2}CH_3$), 3.73 (s, 3H, OCH_3), 2.73 (s, 3H, CH_3), 1.39 (t, J = 7.1 Hz, 3H, -$CH_2\underline{CH_3}$); FT-IR (ύ max, cm^{-1}): 3281 (NH), 3067 (CH aromatic), 2988 (CH aliphatic), 1683 (C=O amide), 1640 (C=N); Anal. Calcd for $C_{24}H_{23}N_5O_5$ (Mwt. : 461): C, 62.46; H, 5.02; N, 15.18; Found: C, 62.55; H, 5.06; N, 15.32.

Ethyl 4-[(4-(3-(3-chlorophenyl)ureido)phenyl)amino]-6-methylfuro[2,3-d]pyrimidine-5-carboxylate (15d):

The titled compound was separated as white crystals (0.35 g, 72%); m.p. 228-232°C; ^{1}HNMR (300 MHz, DMSO-d_6) δ 10.44 (s, 1H, NH D_2O exchangeable), 8.85 (s, 1H, NH D_2O

exchangeable), 8.76 (s, 1H, NH D_2O exchangeable), 8.41(s, 1H, pyrimidine H), 7.71 (s, 1H, ArH), 7.67 (d, *J* = 8.5 Hz, 2H, ArH), 7.47 (d, *J* = 8.5 Hz, 2H, ArH), 7.32 (d, *J* = 8.1 Hz, 1H, ArH), 7.25 (t, *J* = 8.1 Hz, 1H, ArH), 7.01 (d, *J* = 8.1 Hz, 1H, ArH), 4.44 (q, *J* = 7.1 Hz, 2H, -$\underline{CH_2}CH_3$), 2.73 (s, 3H, CH_3), 1.38 (t, *J* = 7.1 Hz, 3H, -$CH_2\underline{CH_3}$); ^{13}C NMR (126 MHz, DMSO-d_6) δ164.13, 160.87, 153.53, 145.69, 141.27, 134.51, 133.16, 130.10, 121.23, 119.86, 118.67, 117.35, 116.39, 113.64, 110.05, 61.81, 14.49, 13.66; FT-IR (ύ max, cm^{-1}): 3288 (NH), 3050 (CH aromatic), 2984 (CH aliphatic), 1685 (C=O amide), 1635 (C=N); MS : (Mwt. : 465) : *m/z* , 467 [M^++2 , (4.4%)] , 465 [M^+, (11.6%)] , 312 (100%); Anal. Calcd for $C_{23}H_{20}ClN_5O_4$: C, 59.29; H, 4.33; N, 15.03; Found: C, 59.39; H, 4.37; N, 15.19.

Ethyl 4-[(4-(3-(4-chlorophenyl)ureido)phenyl)amino]-6-methylfuro[2,3-d]pyrimidine-5-carboxylate (15e):

The titled compound was separated as white crystals (0.104 g, 51%); m.p. 262-265°C; ^{1}HNMR (300 MHz, DMSO-d_6) δ 10.41 (s, 1H, NH D_2O exchangeable), 8.76 (s, 1H, NH D_2O exchangeable), 8.69 (s, 1H, NH D_2O exchangeable), 8.41 (s, 1H, pyrimidine H), 7.67 (d, *J* = 8.5 Hz, 2H, ArH), 7.5 (d, *J* = 8.5 Hz, 2H, ArH), 7.45 (d, *J* = 8.5 Hz, 2H, ArH), 7.32 (d, *J* = 8.5 Hz, 2H, ArH), 4.44 (q, *J* = 7.1 Hz, 2H, -$\underline{CH_2}CH_3$), 2.73 (s, 3H, CH_3), 1.39 (t, *J* = 7.1 Hz, 3H, -$CH_2\underline{CH_3}$); FT-IR (ύ max, cm^{-1}): 3291 (NH), 3122 (CH aromatic), 2988 (CH aliphatic), 1686 (C=O amide), 1640 (C=N); Anal. Calcd for $C_{23}H_{20}ClN_5O_4$ (Mwt. : 465): C, 59.29; H, 4.33; N, 15.03; Found: C, 59.38; H, 4.39; N, 15.14.

Ethyl 4-[(4-(3-(3-chloro-4-methylphenyl)ureido)phenyl)amino]-6-methylfuro[2,3-d]pyrimidine-5-carboxylate (15f):

The titled compound was separated as white crystals (0.247 g, 50%); m.p. 254-256°C; ^{1}HNMR (300 MHz, DMSO-d_6) δ 10.36 (s, 1H, NH D_2O exchangeable), 8.61 (s, 1H, NH D_2O exchangeable), 8.43 (s, 1H, NH D_2O exchangeable), 8.40 (s, 1H, pyrimidine H), 7.77 (s, 1H, ArH), 7.67 (d, *J* = 8.7 Hz, 2H, ArH), 7.46 (d, *J* = 8.7 Hz, 2H, ArH), 7.33 (d, *J* = 7.9 Hz, 1H, ArH), 7.08 (d, *J* = 7.9 Hz, 1H, ArH) , 4.45 (q, *J* = 7.1 Hz, 2H, -$\underline{CH_2}CH_3$), 2.74 (s, 3H, CH_3), 2.25 (s, 3H, Ph-CH_3), 1.40 (t, *J* = 7.1 Hz, 3H, -$CH_2\underline{CH_3}$); FT-IR (ύ max, cm^{-1}): 3284 (NH), 3125 (CH aromatic), 2980 (CH aliphatic), 1682 (C=O amide), 1636 (C=N); Anal. Calcd for $C_{24}H_{22}ClN_5O_4$ (Mwt. : 479): C, 60.06; H, 4.62; N, 14.59; Found: C, 60.23; H, 4.67; N, 14.72.

Ethyl 4-[(4-(3-(3-acetylphenyl)ureido)phenyl)amino]-6-methylfuro[2,3-d]pyrimidine-5-carboxylate (15g):

The titled compound was separated as grey crystals (0.23g, 50%); m.p. 240-244°C; ^{1}HNMR (300 MHz, DMSO-d_6) δ 10.42 (s, 1H , NH D_2O exchangeable), 8.85 (s, 1H, NH D_2O exchangeable), 8.69 (s, 1H, NH D_2O exchangeable), 8.41 (s, 1H, pyrimidine H), 8.07 (s, 1H, ArH), 7.68 (d, *J* = 8.8 Hz, 2H, ArH), 7.58 (d, *J* = 7.9 Hz, 1H, ArH), 7.48 (d, *J* = 8.8 Hz, 2H, ArH), 7.44 (m, 2H, ArH), 4.43 (q, *J* = 7.1 Hz, 2H, -$\underline{CH_2}CH_3$), 2.73 (s, 3H, CH_3), 2.50 (s, 3H,-$COCH_3$), 1.39 (t, *J* = 7.1 Hz, 3H, -$CH_2\underline{CH_3}$); FT-IR (ύ max, cm^{-1}): 3301 (NH), 3130 (CH aromatic), 2988 (CH aliphatic), 1686 (C=O amide), 1643 (C=N); Anal. Calcd for $C_{25}H_{23}N_5O_5$ (Mwt. : 473): C, 63.42; H, 4.90; N, 14.79; Found: C, 63.58; H, 4.92; N, 14.89.

Ethyl 4-[(4-(3-(3-bromophenyl)ureido)phenyl)amino]-6-methylfuro[2,3-d] pyrimidine-5-carboxylate (15h):

The titled compound was separated as white crystals (0.404 g, 75%); m.p. 224-228°C; ^{1}HNMR (300 MHz, DMSO-d_6) δ 10.39 (s, 1H, NH D_2O exchangeable), 8.72 (s, 1H, NH D_2O exchangeable), 8.68 (s, 1H, NH D_2O exchangeable), 8.37(s, 1H, pyrimidine H), 7.84 (s, 1H, ArH), 7.64 (d, *J* = 8.5 Hz, 2H, ArH), 7.43 (d, *J* = 8.5 Hz, 2H, ArH), 7.28 (d, *J* = 8.2 Hz, 1H, ArH), 7.22 (t, *J* = 8.2 Hz, 1H, ArH), 7.11 (d, *J* = 8.2 Hz, 1H, ArH), 4.39 (q, *J* = 7.1 Hz, 2H, -$\underline{CH_2}CH_3$), 2.67 (s, 3H, CH_3), 1.36 (t, *J* = 7.1 Hz, 3H, -$CH_2\underline{CH_3}$); FT-IR (ύ max, cm^{-1}): 3288 (NH), 3047 (CH aromatic), 2990 (CH aliphatic), 1685 (C=O amide), 1632 (C=N); Anal. Calcd for $C_{23}H_{20}BrN_5O_4$ (Mwt : 510): C, 54.13; H, 3.95; N, 13.72; Found: C, 54.37; H, 3.74; N, 13.91.

Ethyl 4-[(4-(3-(4-ethylphenyl)ureido)phenyl)amino]-6-methylfuro[2,3-d] pyrimidine-5-carboxylate (15i):

The titled compound was separated as white crystals (0.311 g, 53%); m.p. 242-246°C; ^{1}HNMR (300 MHz, DMSO-d_6) δ 10.39 (s, 1H, NH D_2O exchangeable), 8.50 (s, 1H, pyrimidine H), 8.39 (s, 2H, 2 NH D_2O exchangeable), 7.65 (d, *J* = 8.9 Hz, 2H, ArH), 7.45 (d, *J* = 8.9 Hz, 2H, ArH), 7.33 (d, *J* = 8.9 Hz, 2H, ArH), 6.84 (d, *J* = 8.9 Hz, 2H, ArH), 4.42 (q, *J* = 7.1 Hz, 2H, -O-$\underline{CH_2}CH_3$), 3.97 (q, *J* = 6.9 Hz, 2H, ph-$\underline{CH_2}CH_3$), 2.71 (s, 3H, CH_3), 1.38 (t, *J* = 7.1 Hz, 3H, -O-$CH_2\underline{CH_3}$), 1.30 (t, *J* = 6.9 Hz, 3H, ph-$CH_2\underline{CH_3}$); FT-IR (ύ max, cm^{-1}): 3285 (NH), 3129 (CH aromatic), 2979 (CH aliphatic), 1692 (C=O amide), 1635 (C=N); Anal. Calcd for $C_{25}H_{25}N_5O_4$ (Mwt. : 459): C, 65.35; H, 5.48; N, 15.24; Found: C, 65.52; H, 5.54; N, 15.32.

Ethyl 6-methyl-4-[4-(3-phenylureido)phenoxy]furo[2,3-d]pyrimidine-5-carboxylate (16a):

The titled compound was separated as buff crystals (0.233 g, 51%); m.p. 183-185°C; ^{1}HNMR (300 MHz, DMSO-d_6) δ 9.23 (s, 1H, NH D_2O exchangeable), 9.16 (s, 1H, NH D_2O exchangeable), 8.49 (s, 1H, pyrimidine H), 7.55 (d, *J* = 8.2 Hz, 2H, ArH), 7.47 (d, *J* = 8.2 Hz, 2H, ArH), 7.28-6.90 (m, 5H, ArH), 4.30 (q, *J* = 7.1 Hz, 2H, -$\underline{CH_2}CH_3$), 2.76 (s, 3H, CH_3), 1.27 (t, *J* = 7.1 Hz, 3H, -$CH_2\underline{CH_3}$); ^{13}C NMR (126 MHz, DMSO-d_6) δ 166.59, 162.51, 161.75, 161.18, 152.75, 146.65, 139.90, 137.34, 128.62, 121.72, 119.26, 118.13, 110.04, 60.58, 13.90, 13.70; FT-IR (ύ max, cm^{-1}): 3314 (NH), 3141 (CH aromatic), 2981 (CH aliphatic), 1661 (C=O amide), 1661 (C=N); MS : (Mwt. : 432) : *m/z* , 433 [M^{+}+1 , (100%)] , 432 [M^{+}, (99.5%)] , 433 (100%); Anal. Calcd for $C_{23}H_{20}N_4O_5$: C, 63.88; H, 4.66; N, 12.96; Found: C, 64.03; H, 4.69; N, 13.07.

Ethyl 6-methyl-4-[4-(3-(m-tolyl)ureido]phenoxy)furo[2,3-d]pyrimidine-5-carboxylate (16b):

The titled compound was separated as green crystals (0.24 g, 52%); m.p. 116-119°C; ^{1}HNMR (300 MHz, DMSO-d_6) δ 9.21 (s, 1H, NH D_2O exchangeable), 9.07 (s, 1H, NH D_2O exchangeable), 8.50 (s, 1H, pyrimidine H), 7.55 (d, *J* = 8.0 Hz, 2H, ArH), 7.33 (s, 1H, ArH), 7.27 (d, *J* = 8.4 Hz, 1H, ArH), 7.14 (t, *J* = 8.4 Hz, 1H, ArH), 7.04 (d, *J* = 8.0 Hz, 2H, ArH), 6.78 (d, *J* = 8.40 Hz, 1H, ArH), 4.31 (q, *J* = 7.1 Hz, 2H, -$\underline{CH_2}CH_3$), 2.74 (s, 3H, CH_3), 2.27 (s, 3H, Ph-CH_3), 1.27 (t, *J* = 7.1 Hz, 3H, -$CH_2\underline{CH_3}$); FT-IR (ύ max, cm^{-1}): 3338 (NH), 3051 (CH aromatic), 2981 (CH aliphatic), 1664 (C=O amide), 1664 (C=N); Anal. Calcd for $C_{24}H_{22}N_4O_5$ (Mwt. : 446): C, 64.57; H, 4.97; N, 12.55; Found: C, 64.61; H, 5.03; N, 12.69.

Ethyl 4-[4-(3-(3-methoxyphenyl)ureido)phenoxy]-6-methylfuro[2,3-d]pyrimidine-5-carboxylate (16c):

The titled compound was separated as buff crystals (0.403 g, 80%); m.p. 170-172°C; ^{1}HNMR (300 MHz, DMSO-d_6) δ 9.02 (s, 1H, NH D_2O exchangeable), 8.97 (s, 1H, NH D_2O exchangeable), 8.49 (s, 1H, pyrimidine H), 7.53 (d, *J* = 8.1 Hz, 2H, ArH), 7.22-7.16 (m, 2H, ArH), 7.14 (d, *J* = 8.1 Hz, 2H, ArH), 6.98 (d, *J* = 8.5 Hz, 1H, ArH), 6.54 (d, *J* = 8.5 Hz, 1H, ArH), 4.32 (q, *J* = 7.1 Hz, 2H, -$\underline{CH_2}CH_3$), 3.73 (s, 3H, OCH_3), 2.76 (s, 3H, CH_3), 1.27 (t, *J* = 7.1 Hz, 3H, -$CH_2\underline{CH_3}$);^{13}C NMR (126 MHz, DMSO-d_6) δ167.14, 163.06, 162.33, 161.75, 160.13, 153.32, 153.20, 147.20, 141.64, 137.77, 129.92, 122.27, 119.87, 111.02, 107.91, 107.57, 104.43, 103.13, 61.15, 55.36, 14.45, 14.26; FT-IR (ύ max, cm^{-1}): 3289 (NH), 3110 (CH aromatic), 2982

(CH aliphatic), 1690 (C=O amide), 1644 (C=N); Anal. Calcd for $C_{24}H_{22}N_4O_6$ (Mwt. : 462): C, 62.33; H, 4.79; N, 12.12; Found: C, 62.41; H, 4.85; N, 12.23.

Ethyl 4-[4-(3-(4-chlorophenyl)ureido)phenoxy]-6-methylfuro[2,3-d]pyrimidine-5-carboxylate (16d):

The titled compound was separated as brown crystals (0.36 g, 75%); m.p. 215-219°C; ^{1}HNMR (300 MHz, DMSO-d_6) δ 9.51 (s, 1H, NH D_2O exchangeable), 8.78 (s, 1H, NH D_2O exchangeable), 8.42 (s, 1H, pyrimidine H), 8.06 (d, *J* = 8.4 Hz, 2H, ArH), 7.89 (d, *J* = 8.4 Hz, 2H, ArH), 7.77 (d, *J* = 8.4 Hz, 2H, ArH), 7.06 (d, *J* = 8.4 Hz, 2H, ArH), 4.44 (q, *J* = 7.1 Hz, 2H, -$\underline{CH_2}CH_3$), 2.63 (s, 3H, CH_3), 1.05 (t, *J* = 7.1 Hz, 3H, -$CH_2\underline{CH_3}$); FT-IR (ύ max, cm^{-1}): 3292 (NH), 3120 (CH aromatic), 2924 (CH aliphatic), 1690 (C=O amide), 1644 (C=N); Anal. Calcd for $C_{23}H_{19}ClN_4O_5$ (Mwt. : 466): C, 59.17; H, 4.10; N, 12.00; Found: C, 59.34; H, 4.08; N, 12.13.

Ethyl 4-[4-(3-(3-chloro-4-methylphenyl)ureido)phenoxy]-6-methylfuro[2,3-d]pyrimidine-5-carboxylate (16e):

The titled compound was separated as brown crystals (0.249 g, 55%); m.p. 203-205°C; ^{1}HNMR (300 MHz, DMSO-d_6) δ 9.52 (s, 1H, NH D_2O exchangeable), 8.49 (s, 1H, NH D_2O exchangeable), 8.40 (s, 1H, pyrimidine H), 7.78 (s, 1H, ArH), 7.55 (d, *J* = 8.7 Hz, 1H, ArH), 7.40 (d, *J* = 8.7 Hz, 1H, ArH), 7.22 (d, *J* = 8.0 Hz, 2H, ArH), 7.13 (d, *J* = 8.0 Hz, 2H, ArH), 4.31 (q, *J* = 7.1 Hz, 2H, -$\underline{CH_2}CH_3$), 2.75 (s, 3H, CH_3), 2.26 (s, 3H, Ph-CH_3), 1.27 (d, *J* = 7.1, Hz, 3H, -$CH_2\underline{CH_3}$); ^{13}C NMR (126 MHz, DMSO-d_6) δ167.12, 163.10, 162.35, 161.70, 153.66, 153.34, 146.93, 140.37, 138.35, 133.42, 131.41, 127.85, 122.10, 119.78, 118.41, 117.30, 107.92, 61.15, 19.25, 14.45, 14.25; FT-IR (ύ max, cm^{-1}): 3338 (NH), 3130 (CH aromatic), 2981 (CH aliphatic), 1690 (C=O amide), 1595 (C=N); Anal. Calcd for $C_{24}H_{21}ClN_4O_5$ (Mwt. : 480): C, 59.94; H, 4.40; N, 11.65; Found: C, 59.99; H, 4.45; N, 11.73.

1-phenyl-3-(4-[(5,6,7,8-tetrahydrobenzo[4,5]thieno[2,3-d]pyrimidin-4-yl)amino]phenyl)urea (20a):

The titled compound was separated as brown crystals (0.11 g, 25%); m.p. 235-236°C; ^{1}HNMR (300 MHz, DMSO-d_6) δ 8.79 (s, 1H, NH D_2O exchangeable), 8.59 (s, 1H, NH D_2O exchangeable), 8.52 (s, 1H, NH D_2O exchangeable), 8.32 (s, 1H, pyrimidine H), 7.44 (d, *J* = 8.0 Hz, 2H, ArH), 7.34 (d, *J* = 8.0 Hz, 2H, ArH), 7.27 (m, *J* = 8.7 Hz, 2H, ArH), 7.15 (d, *J* = 8.7 Hz, 2H, ArH), 6.95 (t, *J* = 8.7 Hz, 1H, ArH), 3.03 (s, 2H, cyclohexyl), 2.82 (s, 2H, cyclohexyl), 1.86

(s, 4H, cyclohexyl); Anal. Calcd for $C_{23}H_{21}N_5OS$ (Mwt. : 415) : C, 66.48; H, 5.09; N, 16.85; S, 7.72; Found: C, 66.62; H, 5.13; N, 16.97; S, 7.86.

1-(4-[(5,6,7,8-Tetrahydrobenzo[4,5]thieno[2,3-d]pyrimidin-4-yl)amino]phenyl)-3-(m-tolyl)urea (20b):

The titled compound was separated as buff crystals (0.20 g, 22%); m.p. >250°C; ^{1}HNMR (300 MHz, DMSO-d_6) δ 8.50 (s, 3H, NH D_2O exchangeable), 8.32 (s, 1H, pyrimidine H), 7.32 (t, *J* = 8.2 Hz, 1H, ArH), 7.22 (d, *J* = 8.4 Hz, 2H, ArH), 7.16 (d, *J* = 8.4 Hz, 2H, ArH), 7.07 (d, *J* = 8.2 Hz, 1H, ArH), 6.77 (s, 1H, ArH), 6.51 (d, *J* = 8.2 Hz, 1H, ArH), 3.12 (s, 2H, cyclohexyl), 2.83 (s, 2H, cyclohexyl), 2.28 (s, 3H, Ph-CH_3), 1.87 (s, 4H, cyclohexyl); FT-IR (ύ max, cm^{-1}): 3299 (NH), 3038 (CH aromatic), 2915 (CH aliphatic), 1682 (C=O amide), 1633 (C=N); Anal. Calcd for $C_{24}H_{23}N_5OS$ (Mwt. : 429): C, 67.11; H, 5.40; N, 16.30; S, 7.47; Found: C, 67.12; H, 5.48; N, 16.39; S, 7.53.

5.1.12.1. 1-(3-Methoxyphenyl)-3-(4-[(5,6,7,8-tetrahydrobenzo[4,5]thieno[2,3-d]pyrimidin-4-yl)amino]phenyl)urea (20c):

The titled compound was separated as brown crystals (0.10 g, 22%); m.p. 206-210°C; ^{1}HNMR (300 MHz, DMSO-d_6) δ 9.37 (s, 1H, NH D_2O exchangeable), 8.89 (s, 1H, NH D_2O exchangeable), 8.78 (s, 1H, NH D_2O exchangeable), 8.43 (s, 1H, pyrimidine H), 7.57 (d, *J* = 8.8 Hz, 2H, ArH), 7.49 (s, 1H, ArH), 7.30 (d, *J* = 8.8, 2H, ArH), 7.18 (t, *J* = 8.1 Hz, 1H, ArH), 6.93 (d, *J* = 8.1 Hz, 1H, ArH), 6.53 (d, *J* = 8.1 Hz, 1H), 3.73 (s, 3H, OCH_3), 3.13 (s, 2H, cyclohexyl), 2.84 (s, 2H, cyclohexyl), 1.85 (s, 4H, cyclohexyl); ^{13}C NMR (126 MHz, DMSO-d_6) δ 159.64, 154.40, 152.63, 152,49, 141.16, 139.80, 137,67, 134.45, 130.86, 129.39, 127.35, 123.71, 118.69, 116.42, 110.34, 107.22, 106.92, 54.86, 25.24, 24.91, 21.75; FT-IR (ύ max, cm^{-1}): 3299 (NH), 3056 (CH aromatic), 2988 (CH aliphatic), 1678 (C=O amide), 1620 (C=N); MS : (Mwt. : 445) : *m/z* , 430 [M^++1 , (3.9%)] , 429 [M^+, (5.4%)] , 149 (100%); Anal. Calcd for $C_{24}H_{23}N_5O_2S$: C, 64.70; H, 5.20; N, 15.72; S, 7.20; Found: C, 64.78; H, 5.24; N, 15.79; S, 7.22.

5.1.12.2. 1-(3-Chlorophenyl)-3-(4-[(5,6,7,8-tetrahydrobenzo[4,5]thieno[2,3-d]pyrimidin-4-yl)amino]phenyl)urea (20d):

The titled compound was separated as buff crystals (0.123 g, 28%); m.p. 275-280°C; ^{1}HNMR (300 MHz, DMSO-d_6) δ 10.11 (s, 1H, NH D_2O exchangeable), 8.82 (s, 1H, NH D_2O exchangeable), 8.62 (s, 1H, NH D_2O exchangeable), 8.43 (s, 1H, pyrimidine H), 7.92 (d, *J* = 8.5

Hz, 2H, ArH), 7.79 (d, J = 8.5 Hz, 2H, ArH), 7.60 (t, J = 8.9, Hz, 1H, ArH), 7.37 (s, 1H, ArH), 6.91 (d, J = 8.9 Hz, 1H, ArH), 6.60 (d, J = 8.9 Hz, 1H, ArH), 3.10 (s, 2H, cyclohexyl), 2.81 (s, 2H, cyclohexyl), 1.84 (s, 4H, cyclohexyl).

1-(4-Chlorophenyl)-3-(4-((5,6,7,8-tetrahydrobenzo[4,5]thieno[2,3-d]pyrimidin-4-yl)amino]phenyl)urea (20e):

The titled compound was separated as brown crystals (0.11 g, 26%); m.p. >300°C; ^{1}HNMR (300 MHz, DMSO-d_6) δ 10.04 (s, 1H, NH D_2O exchangeable), 8.78 (s, 1H, NH D_2O exchangeable), 8.60 (s, 1H, NH D_2O exchangeable), 8.38 (s, 1H, pyrimidine H), 7.47 (d, J = 8.7 Hz, 2H, ArH), 7.35 (d, J = 8.7Hz, 2H, ArH), 7.32 (d, J = 9.0 Hz, 2H, ArH), 7.29 (d, J = 9.0 Hz, 2H, ArH), 3.06 (s, 2H, cyclohexyl), 2.78 (s, 2H, cyclohexyl), 1.82 (s, 4H, cyclohexyl).

1-(3-Chloro-4-methylphenyl)-3-(4-[(5,6,7,8-tetrahydrobenzo[4,5]thieno[2,3-d]pyrimidin-4-yl)amino]phenyl)urea (20f):

The titled compound was separated as buff crystals (0.109 g, 23%); m.p. >300°C; ^{1}HNMR (300 MHz, DMSO-d_6) δ 8.79 (s, 1H, NH D_2O exchangeable), 8.64 (s, 1H, NH D_2O exchangeable), 8.54 (s, 1H, NH D_2O exchangeable), 8.36 (s, 1H, pyrimidine H), 7.76 (s, 1H, ArH), 7.32 (d, J = 8.4 Hz, 2H, ArH), 7.18 (d, J = 8.4 Hz, 2H, ArH), 7.06 (d, J = 8.1 Hz, 1H, ArH), 6.55 (d, J = 8.1 Hz, 1H, ArH) , 3.10 (s, 2H, cyclohexyl), 2.83 (s, 2H, cyclohexyl), 2.25 (s, 3H, Ph-CH_3), 1.86 (s, 4H, cyclohexyl).

1-(3-Acetylphenyl)-3-(4-[(5,6,7,8-tetrahydrobenzo[4,5]thieno[2,3-d]pyrimidin-4-yl)amino]phenyl)urea (20g):

The titled compound was separated as grey crystals (0.144 g, 30%); m.p. 265-269°C; ^{1}HNMR (300 MHz, DMSO-d_6) δ 10.01 (s, 1H, NH D_2O exchangeable), 8.87 (s, 1H, NH D_2O exchangeable), 8.61 (s, 1H, NH D_2O exchangeable), 8.56 (s, 1H, pyrimidine H), 8.06 (s, 1H, ArH), 7.67(d, J = 8.7 Hz, 2H, ArH), 7.56 (d, J = 8.7 Hz, 2H, ArH), 7.45 – 7.38 (m, 3H, ArH), 3.91 (s, 2H, cyclohexyl), 2.83 (s, 2H, cyclohexyl), 2.57 (s, 3H, $COCH_3$), 1.86 (s, 4H, cyclohexyl).

1-Phenyl-3-(4-[(5,6,7,8-tetrahydrobenzo[4,5]thieno[2,3-d]pyrimidin-4 yl)oxy]phenyl)urea (21a):

The titled compound was separated as grey crystals (0.176 g, 44%); m.p. 197-200°C; ^{1}HNMR (300 MHz, DMSO-d_6) δ 8.82 (s, 1H, NH D_2O exchangeable), 8.74 (s, 1H, NH D_2O

exchangeable), 8.47 (s, 1H, pyrimidine H), 7.53 (d, *J* = 8.7 Hz, 2H, ArH), 7.47 (d, *J* = 8.7 Hz, 2H, ArH), 7.33 – 6.92 (m, 5H, ArH), 3.03 (s, 2H, cyclohexyl), 2.87 (s, 2H, cyclohexyl), 1.87 (s, 4H, cyclohexyl); FT-IR (ύ max, cm^{-1}): 3302 (NH), 3028 (CH aromatic), 2942 (CH aliphatic), 1690 (C=O amide), 1643 (C=N); Anal. Calcd for $C_{23}H_{20}N_4O_2S$ (Mwt. : 416): C, 66.33; H, 4.84; N, 13.45; S, 7.70; Found: C, 66.41; H, 4.88; N, 15.62; S, 7.71.

1-(4-[(5,6,7,8-Tetrahydrobenzo[4,5]thieno[2,3-d]pyrimidin-4-yl)oxy]phenyl)-3-(m-tolyl)urea (21b):

The titled compound was separated as grey crystals (0.21 g, 46%); m.p. 214-216°C; ^{1}HNMR (300 MHz, DMSO-d_6) δ 8.73 (s, 1H, NH D_2O exchangeable), 8.59 (s, 1H, NH D_2O exchangeable), 8.47 (s, 1H, pyrimidine H), 7.57 (d, *J* = 8.4 Hz, 2H, ArH), 7.34 (s, 1H, ArH), 7.30 (d, *J* = 8.4 Hz, 1H, ArH), 7.20 (t, *J* = 8.4 Hz, 1H, ArH), 7.17 (d, *J* = 8.4 Hz, 2H, ArH), 6.84 (d, *J* = 8.4 Hz, 1H, ArH), 3.03 (s, 2H, cyclohexyl), 2.88 (s, 2H, cyclohexyl), 2.28 (s, 3H, Ph-CH_3), 1.87 (s, 4H, cyclohexyl); ^{13}C NMR (126 MHz, DMSO-d_6) δ 167.52, 162.99, 152.53, 151.97, 146.44, 139.57, 137.86, 137.19, 135.52, 128.53, 126.88, 122.51, 122.13, 120.77, 118.70, 115.37, 25.42, 24.93, 22.33, 21.16; FT-IR (ύ max, cm^{-1}): 3302 (NH), 3038 (CH aromatic), 2945 (CH aliphatic), 1682 (C=O amide), 1625 (C=N); MS : (Mwt. : 430) : *m/z* , 431 [M^++1 , (59.6%)] , 430 [M^+, (78.9%)] , 300 (100%); Anal. Calcd for $C_{24}H_{22}N_4O_2S$: C, 66.96; H, 5.15; N, 13.01; S, 7.45; Found: C, 67.08; H, 5.17; N, 13.14; S, 7.52.

1-(3-Methoxyphenyl)-3-(4-[(5,6,7,8-tetrahydrobenzo[4,5]thieno[2,3-d]pyrimidin-4-yl)oxy]phenyl)urea (21c):

The titled compound was separated as green crystals (0.303 g, 62%); m.p. 216-220°C; ^{1}HNMR (300 MHz, DMSO-d_6) δ 8.73 (s, 1H, NH D_2O exchangeable), 8.59 (s, 1H, NH D_2O exchangeable), 8.47 (s, 1H, pyrimidine H), 7.54 (d, *J* = 8.4 Hz, 2H, ArH), 7.22 (t, *J* = 8.0 Hz, 1H, ArH) , 7.15 (d, *J* = 8.4 Hz, 2H, ArH), 7.11 (s, 1H, ArH), 6.95 (d, *J* = 8.0 Hz, 1H, ArH), 6.49 (d, *J* = 8.0 Hz, 1H, ArH), 3.75 (s, 3H, OCH_3), 3.02 (s, 2H, cyclohexyl), 2.87 (s, 2H, cyclohexyl), 1.86 (s, 4H, cyclohexyl); ^{13}C NMR (126 MHz, DMSO-d_6) δ 167.69, 164.20, 160.24, 159.90, 152.71, 149.86, 149.28, 142.62, 135.55, 128.56, 127.56, 121.12, 120.58, 118.84, 111.70, 104.06, 102.80, 54.94, 26.05, 25.45, 22.87, 22.31; FT-IR (ύ max, cm^{-1}): 3230 (NH), 3094 (CH aromatic), 2943 (CH aliphatic), 1688 (C=O amide), 1620 (C=N); Anal. Calcd for $C_{24}H_{22}N_4O_3S$ (Mwt. : 446): C, 64.56; H, 4.97; N, 12.55; S, 7.18; Found: C, 64.65; H, 4.99; N, 12.63; S, 7.22.

1-(4-Chlorophenyl)-3-(4-[(5,6,7,8-tetrahydrobenzo[4,5]thieno[2,3-d]pyrimidin-4-yl)oxy]phenyl)urea (21d):

The titled compound was separated as brown crystals (0.311 g, 60%); m.p. >300°C; 1HNMR (300 MHz, DMSO-d6) δ 8.59 (s, 1H, NH D_2O exchangeable), 8.45 (s, 1H, pyrimidine H), 7.80 (s, 1H, NH D_2O exchangeable), 7.51 (d, *J* = 8.0 Hz, 2H, ArH), 7.43 (d, *J* = 8.0 Hz, 2H, ArH), 6.95 (d, *J* = 8.4 Hz, 2H, ArH), 6.89 (d, *J* = 8.4 Hz, 2H, ArH), 3.02 (s, 2H, cyclohexyl), 2.87 (s, 2H, cyclohexyl), 1.86 (s, 4H, cyclohexyl); FT-IR (ύ max, cm^{-1}): 3292 (NH), 3110 (CH aromatic), 2924 (CH aliphatic), 1680 (C=O amide), 1644 (C=N); Anal. Calcd for $C_{23}H_{19}ClN_4O_2S$ (Mwt. : 450): C, 61.26; H, 4.25; N, 12.42; S, 7.11; Found: C, 61.41; H, 4.32; N, 12.49; S, 7.24.

1-(3-Chloro-4-methylphenyl)-3-(4-[(5,6,7,8 tetrahydrobenzo[4,5]thieno[2,3-d]pyrimidin-4-yl)oxy]phenyl)urea (21e):

The titled compound was separated as grey crystals (0.306 g, 72%); m.p. 237-240°C; ^{1}HNMR (300 MHz, DMSO-d_6) δ 8.73 (s, 1H, NH D_2O exchangeable), 8.59 (s, 1H, NH D_2O exchangeable), 8.47 (s, 1H, pyrimidine H), 7.71 (s, 1H, ArH), 7.55 (d, *J* = 8.9 Hz, 2H, ArH), 7.29 – 7.21 (m, 2H, ArH), 7.19 (d, *J* = 8.9 Hz, 2H, ArH), 3.04 (s, 2H, cyclohexyl), 2.88 (s, 2H, cyclohexyl), 2.27 (s, 3H, Ph-CH_3), 1.88 (s, 4H, cyclohexyl); ^{13}C NMR (126 MHz, DMSO-d_6) δ167.75, 164.09, 155.29, 152.68, 151.25, 145.91, 143.22, 135.65, 132.80, 130.29, 127.54, 121.39, 121.21, 119.82, 119.35, 119.00, 118.84, 26.03, 25.45, 22.86, 19.23; FT-IR (ύ max, cm^{-1}): 3293 (NH), 3090 (CH aromatic), 2927 (CH aliphatic), 1689 (C=O amide), 1647 (C=N); Anal. Calcd for $C_{24}H_{21}ClN_4O_2S$ (Mwt. : 464): C, 62.00; H, 4.55; N, 12.05; S, 6.90; Found: C, 62.09; H, 4.59; N, 12.18; S, 6.98.

Biological Evaluation assay

In vitro VEGFR-2 tyrosine kinase activity

Assay protocol

The assay was performed using Kinase-Glo Plus luminescence kinase assay kit (Promega). It measures kinase activity by quantitating the amount of ATP remaining in solution following a kinase reaction. The luminescent signal from the assay is correlated with the amount of ATP present and is inversely correlated with the amount of kinase activity. The compounds were diluted to 100 µl in 10% DMSO and 5 µl of the dilution was added to a 50 µl reaction so that the final concentration of DMSO is 1% in all of reactions. All of the enzymatic reactions were conducted at 30 ^{o}C for 40 minutes. The 50 µl reaction mixture contains 40 mM Tris, pH 7.4, 10

mM $MgCl_2$, 0.1 mg/ml BSA, 1 mM DTT, 10 µM ATP, Kinase substrate and VEGFR. After the enzymatic reaction, 50 µl of Kinase-Glo Plus Luminescence kinase assay solution (Promega) was added to each reaction and incubate the plate for 5 minutes at room temperature. Luminescence signal was measured using a BioTek Synergy 2 microplate reader. VEGFR activity assays were performed in duplicate at each concentration.

Data analysis

The luminescence data were analyzed using the computer software, Graphpad Prism. The difference between luminescence intensities in the absence of VEGFR (Lu_t) and in the presence of VEGFR (Lu_c) was defined as 100 % activity ($Lu_t – Lu_c$). Using luminescence signal (Lu) in the presence of the compound, % activity was calculated as:

% activity = $\{(Lu_t - Lu)/(Lu_t - Lu_c)\}\times100\%$, where Lu= the luminescence intensity in the presence of the compound. % Inhibition was calculated as: % inhibition = 100 (%) - % activity.

IC_{50} determination for inhibitor against VEGFR was calculated. The values of % activity versus a series of compound concentrations (1 nM - 10 nM - 100 nM - 1 µM - 10 µM) were then plotted using non-linear regression analysis of Sigmoidal dose-response curve generated with the equation $Y=B+(T-B)/1+10^{((LogEC50-X)\times Hill\ Slope)}$, where Y=percent activity, B=minimum percent activity, T=maximum percent activity, X= logarithm of compound and Hill Slope=slope factor or Hill coefficient. The IC_{50} value was determined by the concentration causing a half-maximal percent activity.

In vitro multiple-kinase inhibition assay

Assay protocol

The assay was performed using the same conditions for VEGFR-2 activity assay (section 5.2.1.1). The assays for c-Kit was an exception, it was performed using ADP-Glo Kinase assay reagents (Promega). It measures kinase activity by quantitating the ADP amount produced from the enzymatic reaction. The luminescent signal from the assay is correlated with the amount of ADP present and is directly correlated with the amount of kinase activity. Other conditions are same to the previous section except the final reaction volume was reduced to 25 µl. After the 40 min kinase reaction at 30 ^oC, 25 µl of ADP-Glo reagent was added and incubated for 45 min at room temperature followed by another 50 min incubation with 50 µl of Kinase detection mixture. Luminescence signal was measured using a BioTek *Synergy 2* microplate reader

Data analysis

The luminescence data were analyzed using the same computer software and the same protocol used for VEGFR-2 activity assay (section 5.2.1.2)

In vitro HUVEC Anti-proliferative assay

Assay protocol

Doxorubicin, a known cancer treatment, is used as control compound. Doxorubicin concentration is 0.0033-33.3 µM, 3 folds dilutions of the tested compounds. HUVEC cells were cultured in Medium 200 with 2% LVES and 1% Pen-strep. To perform the proliferation assay, HUVEC cells were seeded at 5000 cells/ 50µl /well in a 96-well black clear-bottom tissue culture plate. Cells were incubated at 37°C and 5% CO_2 overnight to allow them to recover and reattach. Dilutions are made in growth medium so that the final concentration of DMSO is 0.1% in all treatments.

Next day cells were treated with test compounds for 72 hours. After treatment, cell proliferation was measured by Fluorescent quantitation of alamarBlue reagent. The alamarBlue assay incorporates a fluorometric/colorimetric growth indicator based on detection of metabolic activity. Specifically, resazurin, the active ingredient in the alamarBlue reagent, is blue in color and virtually non-fluorescent. Upon entering cells, resazurin is reduced to resorufin, a compound that is red in color and highly fluorescent. Continued cell growth maintains a reduced environment, therefore increasing the overall fluorescence and color of the media surrounding cells. The fluorescence intensity of alamarBlue reagent is shown to be directly proportional to cell number. To perform the alamarBlue assay, 10 µl of alamarBlue reagent was added to each well and the plate was incubated at 37°C for an additional 2 hours. Fluorescence intensity was measured at an excitation of 530 nm and an emission of 590 nm using a BioTek Synergy™ 2 microplate reader.

Data analysis

Cell proliferation assays were performed in triplicate at each concentration. The fluorescent intensity data were analyzed using the computer software, Graphpad Prism. In the absence of the compound, the fluorescent intensity (F_t) in each data set was defined as 100%. In the absence of cells, the fluorescent intensity (F_b) in each data set was defined as 0%. The percent cell in the presence of each compound was calculated according to the following equation: %cell = (F-

F_b)/(F_t-F_b), where F= the fluorescent intensity in the presence of the compound, F_b= the fluorescent intensity in the absence of cells, and F_t = the fluorescent intensity in the absence of the compound.

The values of % cell versus a series of compound concentrations were then plotted using non-linear regression analysis of Sigmoidal dose-response curve generated with the equation $Y=B+(T-B)/1+10^{((LogEC50-X)\times Hill\ Slope)}$, where Y=percent cell, B=minimum percent cell, T=maximum percent cell, X= logarithm of compound and Hill Slope=slope factor or Hill coefficient.

Histopathological Examination

Autopsy samples were taken from subcutaneously transplanted EAC, liver, kidney, heart and brain of mice in different groups and fixed in 10% formol saline for 24 hours. Washing was carried out with tap water then serial dilutions of alcohol (methyl, ethyl and absolute ethyl) were used for dehydration. Specimens were cleared in xylene and embedded in paraffin at 56°C in hot air oven for 24 hours. Paraffin beeswax tissue blocks were prepared for sectioning at 4 µm thickness by slidge microtome. The obtained tissue sections were collected on glass slides, deparaffinized, stained by hematoxylin & eosin stain for routine examination by light electric microscopy.[1]

Immunohistochemistry

Paraffin embedded EAC sections of 3 µm thickness were first rehydrated in xylene and then in graded ethanol solutions. The slides were then blocked with 5% bovine serum albumin (BSA) in tris buffered saline (TBS) for 2 h. The sections were then immunostained with primary antibodies specific to phospho-VEGFR-2 (Tyr951), CD31, CD34 and active caspase 3 at a concentration of 1 µg/ml containing 5% BSA in TBS and incubated overnight at 4 °C. After washing the slides with TBS, the sections were incubated with goat anti-rabbit secondary antibody. Sections were then washed with TBS and incubated for 5–10 min in a solution of 0.02% diaminobenzidine containing 0.01% H_2O_2. Counter staining was performed using hematoxylin, and slides were visualized using a light microscope. Fraction of DAB-positive immunoreactive area was automatically calculated in high power fields (100x) as area percentage of immunopositive cells to the total area of the microscopic field using a digital video camera mounted on a light microscope (CX21, OLYMPUS, JAPAN). Percent of microvessel density (%

MVD) was obtained by automatically calculating fraction of CD31 and CD34 DAB-positive immunoreactive area in high power fields (100x) as area percentage of immunopositive cells to the total area of the microscopic field. All steps for immunohistochemical evaluation were carried out using image analysis software (Image J, 1.46a, NIH, USA) according to the method described by Mezei et al. [2]

In vitro Anti-proliferative activity against 60 cell line panel

Assay protocol

The human tumor cell lines of the cancer screening panel are grown in RPMI 1640 medium containing 5% fetal bovine serum and 2 mM L-glutamine. For a typical screening experiment, cells are inoculated into 96 well microtiter plates in 100 µL at plating densities ranging from 5,000 to 40,000 cells/well depending on the doubling time of individual cell lines. After cell inoculation, the microtiter plates are incubated at 37° C, 5 % CO_2, 95 % air and 100 % relative humidity for 24 hours prior to addition of experimental drugs. After 24 hours, two plates of each cell line are fixed *in situ* with TCA, to represent a measurement of the cell population for each cell line at the time of drug addition (Tz). Experimental drugs are solubilized in dimethyl sulfoxide at 400-fold the desired final maximum test concentration and stored frozen prior to use. At the time of drug addition, an aliquot of frozen concentrate is thawed and diluted to twice the desired final maximum test concentration with complete medium containing 50 µg/mL gentamicin. Additional four, 10-fold or ½ log serial dilutions are made to provide a total of five drug concentrations plus control. Aliquots of 100 µl of these different drug dilutions are added to the appropriate microtiter wells already containing 100 µl of medium, resulting in the required final drug concentrations. Following drug addition, the plates are incubated for an additional 48 hrs at 37°C, 5 % CO_2, 95 % air, and 100 % relative humidity. For adherent cells, the assay is terminated by the addition of cold TCA. Cells are fixed *in situ* by the gentle addition of 50 µl of cold 50 % (w/v) TCA (final concentration, 10 % TCA) and incubated for 60 minutes at 4°C. The supernatant is discarded, and the plates are washed five times with tap water and air dried. Sulforhodamine B (SRB) solution (100 µl) at 0.4 % (w/v) in 1 % acetic acid is added to each well, and plates are incubated for 10 minutes at room temperature. After staining, unbound dye is removed by washing five times with 1 % acetic acid and the plates are air dried. Bound stain is subsequently solubilized with 10 mM trizma base, and the absorbance is read on an automated

plate reader at a wavelength of 515 nm. For suspension cells, the methodology is the same except that the assay is terminated by fixing settled cells at the bottom of the wells by gently adding 50 µl of 80 % TCA (final concentration, 16 % TCA).

Data analysis

Using the seven absorbance measurements [time zero, (Tz), control growth, (C), and test growth in the presence of drug at the five concentration levels (Ti)], the percentage growth is calculated at each of the drug concentrations levels. Percentage growth inhibition is calculated as:[(Ti-Tz)/(C-Tz)] x 100 for concentrations for whichTi>/ = Tz and [(Ti - Tz)/Tz] x 100 for concentrations for which Ti < Tz.[3]

References:

1. Banchroft, S.A., Turner, D.R. *Theory and practice of histological technique*, (ed. John, B., Maryline, G.) (Churchil Livingstone, New York, London, San Francisco, Tokyo, 1996).
2. Mezei, T., Szakács, M., Dénes, L., Jung, J. & Egyed-Zsigmond, I. Semiautomated image analysis of high contrast tissue areas using hue/saturation/brightness based color filtering. *Acta Medica Marisiensis* **57**, 679-684 (2011).
3. Boyd, M.R. & Paull, K.D. Some practical considerations and applications of the national cancer institute in vitro anticancer drug discovery screen. *Drug Develop Res* **34**, 91-109 (1995).
